# A nationwide survey on the use of heated humidified high flow oxygen therapy on the paediatric wards in the UK: current practice and research priorities

**DOI:** 10.1186/s12887-020-1998-1

**Published:** 2020-03-06

**Authors:** Osama Hosheh, Christopher T. Edwards, Padmanabhan Ramnarayan

**Affiliations:** 1 Paediatric Intensive Care Unit, Birmingham Children’s Hospital, Birmingham Women’s and Children’s Hospital Foundation Trust, Steelhouse Lane, Birmingham, B4 6NH UK; 2 Leeds Regional Paediatric Respiratory & Cystic Fibrosis Centre, Leeds Children’s Hospital, Leeds General Infirmary, Leeds, UK; 3 Paediatric Intensive Care Unit, St Mary’s Hospital, Imperial College Healthcare NHS Trust, London, UK; 4 Children’s Acute Transport Service, Great Ormond Street Hospital NHS Foundation Trust, London, UK

**Keywords:** High flow nasal cannula, Paediatrics, Children, Oxygen therapy, Adjunctive therapy

## Abstract

**Background:**

Heated Humidified High Flow Nasal Cannula Oxygen Therapy (HHFNC) is increasingly used on the paediatric wards and High Dependency Units (HDU) for different types of pathologies and different age groups. We aimed to describe current practice related to the use of HHFNC on the paediatric wards and HDUs, weaning practices and preferred outcome measures for future research.

**Methods:**

We carried out a cross-sectional online survey of UK paediatric consultants or their delegates working on the paediatric wards. Descriptive analysis of their geographical, and organizational characteristics, their specialties, and their level of experience was investigated. Reasons for HHFNC initiation, weaning criteria, patients’ characteristics and their primary pathologies were also analysed.

**Results:**

Participation of 218 paediatricians from 81 hospitals (Median: 2.7, Range: 1–11) was registered. HHFNC was provided in most of the surveyed hospitals (93%, 75/81). A High Dependency Unit (HDU) was available in 47 hospitals (58%); less than a third of those have a dedicated paediatrician. Decisions around HHFNC were made solely by paediatricians in (75%) of the cases, mostly at hospitals with no HDU compared to those with dedicated HDUs (70.3% VS 36.6, 95%CI:22.6–50.4%, *P* < .001). HHFNC was reported by nearly two-thirds (68%) of the practitioners who used it on the wards to be as effective or superior to CPAP (Continuous Positive Airway Pressure) with fewer complications. Failure rate while on HHFNC was identified as the most important outcome measure in any future research followed by the length of need for HHFNC support (37.1, and 28% respectively).

**Conclusion:**

This survey showed support for developing paediatric-specific national guidance on the use of HHFNC on the wards. Our list of defined research priorities may help guide further collaborative research efforts in this field.

## Background

Heated Humidified High Flow Nasal Cannula Oxygen Therapy (HHFNC) has become increasingly popular as an option for non-invasive respiratory support of infants and children in critical care [1]. More recently, HHFNC has also been introduced into paediatric wards in the United Kingdom (UK), mainly for the management of bronchiolitis [2], and although high-quality evidence has begun to emerge on the clinical effectiveness of HHFNC compared to standard low flow oxygen therapy [3], other trials that compared it with CPAP were primarily bronchiolitis-focused [4] with debatable interpretations [1, 5]. The current use of HHFNC in infants and children, therefore, is still largely based on individual experience with a clear lack of national and international guidance.

The primary mechanism of action for HHFNC is not well known but has many theoretical ones by which it reduces the work of breathing and improves efficiency of ventilation [6, 7] by washing out the nasopharyngeal dead space leading to improved alveolar ventilation, reduction in the inspiratory resistance associated with the nasopharynx, improvement in conductance and pulmonary compliance by supplying adequately warmed and humidified gas and provision of positive distending pressure for lung recruitment although the latest is variable [1, 8]. Optimal starting flow rate, strategies for weaning, feeding, and use of adjunctive therapy such as nebuliser therapy are some of the unanswered questions while applying HHFNC [9]. Moreover, its use for diseases other than bronchiolitis [10], and during paediatric retrieval is still being explored [11]. Regarding flow rates, there are no national or international guidelines in infants or children yet [12, 13] and although a range of 1.5–2 L/kg/min has widely been adopted in current paediatric practice and previous clinical studies, we expect that there is a spectrum of maximum HHFNC flow rates that are currently trialled on our paediatric wards. In addition, there are no solid weaning protocols for HHFNC which may prolong the length of hospital stay [10, 14, 15]. There is also a limited number of observational studies describing the supportive care of patients receiving HHFNC (i.e. nasogastric (NGT) or nasojejunal tubes (NJT) VS. oral feeding, aerosol delivery techniques for inhalational drug delivery, and use of sedation while on HHFNC) [9, 10].

Because of the uncertainty surrounding the use of HHFNC in paediatric practice, we aimed to survey the UK paediatricians with the following objectives: a) to describe the current practice related to the use of HHFNC on the paediatric wards and HDUs for different age groups and different pathologies; b) to describe weaning practices and supportive care during HHFNC, and c) to define research priorities and preferred outcome measures for any future randomised controlled trials.

## Methods

We carried out a cross-sectional online survey of UK paediatric consultants using the Online Survey Software (formerly BOS, onlinesurveys.ac.uk, Jisc software, UK) to elicit their responses regarding practice related to HHFNC and their perceptions regarding research priorities. The survey covered four main domains:

1. general information about the respondents,
2. their wards and their patients’ characteristics including their primary illnesses,
3. information about the use of HHFNC in practice including responses to two case scenarios, comparison with both low flow oxygen therapy (LF) and CPAP.
4. finally, the respondents’ opinion of future trials on HHFNC and the key priorities in any further research.

An initial version of the survey was piloted (by 7 paediatric consultants) in three regions across the UK and these responses were used to inform the final questionnaire used in this survey (Additional file 1, Survey questionnaire). Questions were directed to paediatric consultants (or their delegates such as a senior registrar or a nurse practitioner) who spend more than 50% of their clinical time in their specialty within the UK. Completion of the survey was voluntary, and consent was not required. The survey link was initially distributed by the Royal College of Paediatrics and Child Health (RCPCH) e-bulletin, and by the British Paediatric Respiratory Society (BPRS) through their mailing lists. We also requested the 12 regional Paediatric Intensive Care retrieval services to forward the survey link to the acute hospitals in their geographical area. Phone calls to the paediatric wards and HDUs were also made where their regions were noticed to be underrepresented in the survey. Data were collected between September 2018 and June 2019.

Data about the number of hospitals with paediatric services in the UK were obtained from the RCPCH Medical Force Census 2015 [16]. The definition of HDU is detailed in (High Dependency Care for Children, Time to Move On, RCPCH, October 2014). High Dependency Care (HDC) describes the child who is critically ill requiring enhanced observation, monitoring and intervention but also is used to describe the child who is not critically ill but requires additional nursing care for other reasons [17].

We primarily used the respondent as the unit of analysis other than for questions relating to hospital characteristics. Results are reported as proportions and/or means as appropriate. Significance testing for differences in proportions was performed using the chi-square test and for differences in means for normally distributed data using the Student t-test. Data analyses were performed using Stata v16 (Stata Corporation, College Station, USA) and Microsoft Excel 2016 (Microsoft Corporation, USA).

## Results

**Fig. 1:**
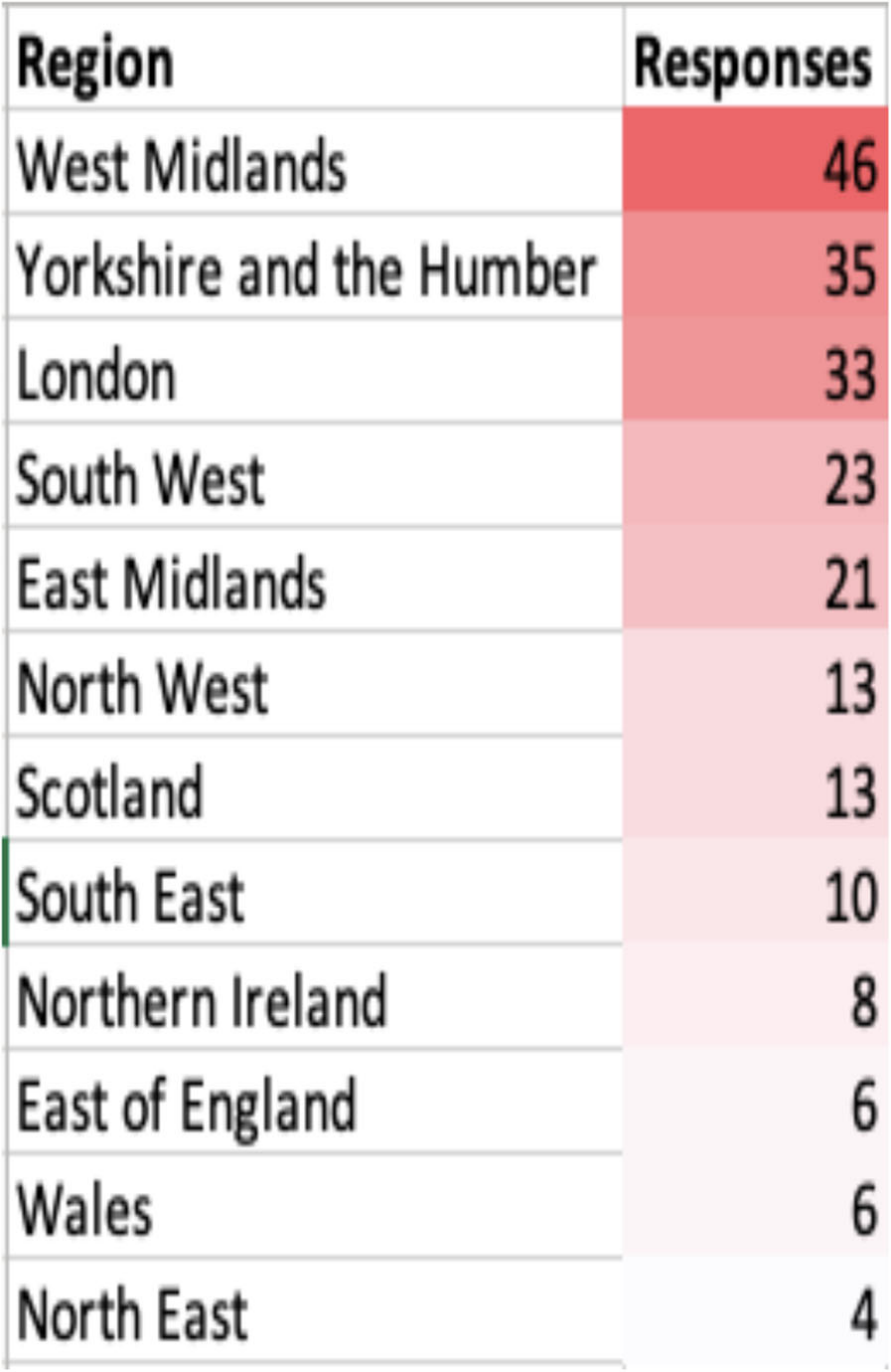
Respondents per region

A total of 218 respondents participated in the survey, representing 81 hospitals across the 12 regions in the UK (total registered hospitals with acute paediatric services in Great Britain and Northern Ireland as per the RCPCH Medical Force Census 2015 were 171) (Fig. 1). The median response was 2.7 (range: 1–11) per hospital. Majority of the participants were paediatric consultants (213/218, 97.7%) with a wide range of clinical experience in their field (Table 1).

**Table 1:** Characteristics of the respondents by specialty and experience

	Working in hospitals with HDU (*n* = 164, %)^c^	Working in hospitals with no HDU (*n* = 54, %)^c^	*p*-value
Main Specialty (> 50% clinical time)			
General Paediatrics	119 (72.6)	48 (89)	.0137
PEM^a^	5 (3)	1 (1.8)	.63
PICM^b^	4 (2.4)	0	
Cardiology	5 (3)	1 (1.8)	.63
Respiratory	25 (15.2)	1 (1.8)	.008
Neonates	2 (1.2)	2 (3.7)	.23
Others (HDU consultant, nurse practitioner)	4 (2.4)	1 (1.8)	.79
Clinical Experience			
< 1 Year	10 (6)	2 (3.7)	.5
1–5 Years	60 (36.6)	20 (37)	.95
6–10 Years	54 (32.9)	14 (8.5)	.0005
> 10 Years	40 (24.4)	18 (10.9)	.035

^a^Paediatric Emergency Medicine, ^b^Paediatric Intensive Care Medicine. ^c^Rounded percentages where possible

Forty-seven hospitals (58%) had dedicated HDUs, with more than a third (19/47, 40%) were with an on-site Paediatric Intensive Care support (PICU). Twelve HDUs (12/19, 63%) were solely managed by the intensive care team and only seven HDUs (7/47 ≈ 15%) had a dedicated paediatrician. The majority of HDUs provided all types of non-invasive ventilation (including Bilevel Positive pressure ventilation, BLPAP) and long-term invasive ventilation (LTV) (43/47, 91.5%). Table 2 represents patient categories that were generally managed on hospital wards.

**Table 2:** Categories of paediatric patients admitted to the wards/HDU in 81 hospitals

Patient Categories on the Ward/HDU	Respondent (n^a^, %)	Hospital (n^a^, %)
Medical	207 (95)	78 (96)
Respiratory	188 (86.2)	76 (94)
Surgical	162 (74.3)	65 (80)
Neonates < 28 days	150 (68.8)	61 (75)
Neurology/Neurosurgery	126 (57.8)	51 (63)
Trauma	121 (55.5)	49 (60)
Cardiac/Cardiac Surgical	55 (25.2)	26 (32)
Others (ENT, Plastics, Burns, Gastro)	18 (8.3)	12 (15)

^a^Based on 218 responses, and 81 hospitals

### Use of HHFNC

**Fig. 2:**
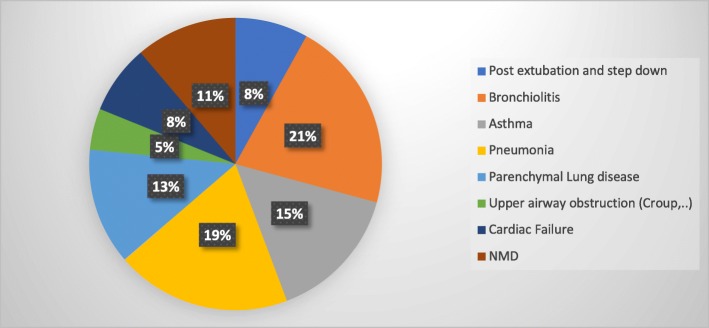
Initiation and modification of HHFNC (NMD: Neuromuscular diseases)

Respondents reported using HHFNC in a variety of illnesses on their wards particularly where HDU and intensive care facilities are not readily available (such as respiratory, cardiac, and neuromuscular diseases). Respiratory diseases collectively accounted for more than 75% of the reasons to start HHFNC (Fig. 2).

**Fig. 3:**
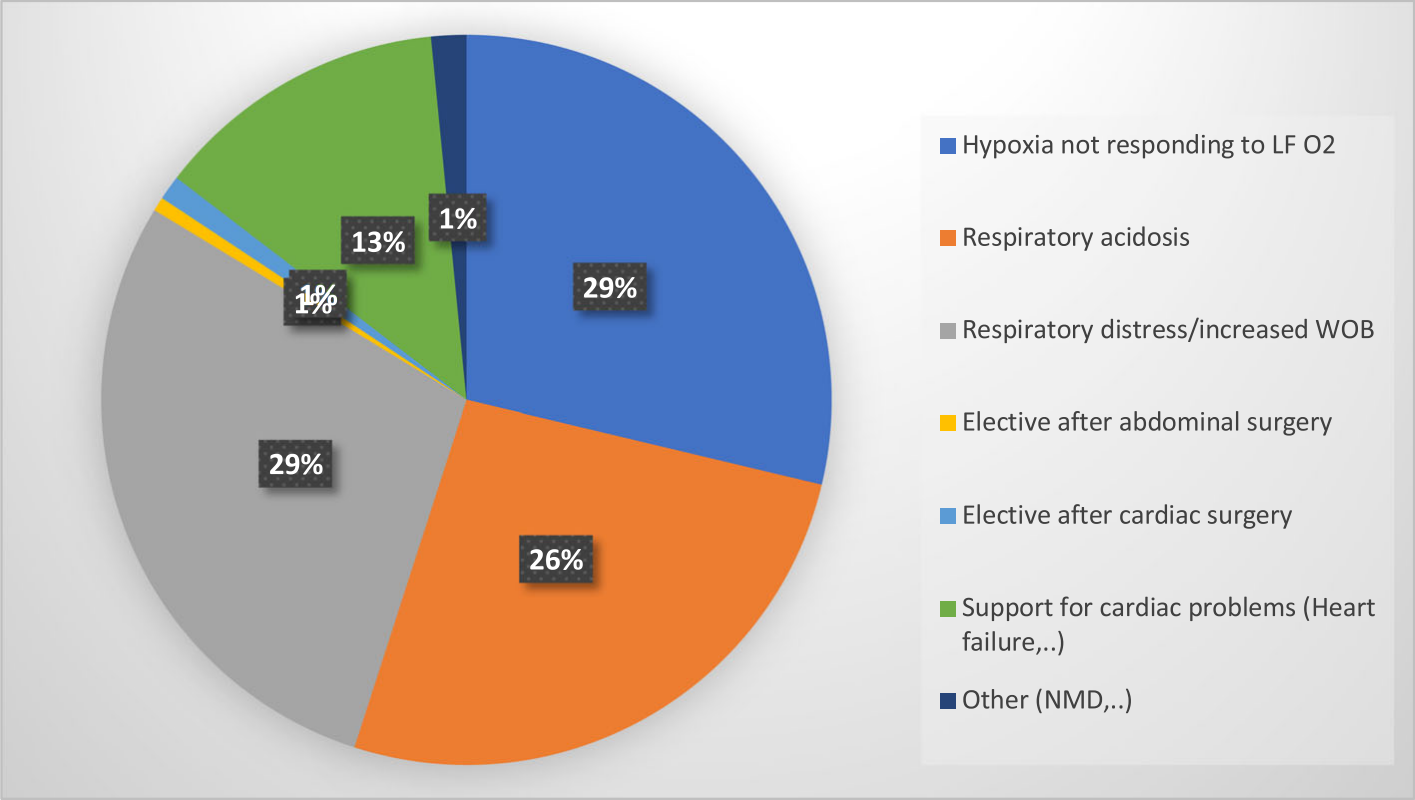
Indications for HHFNC on the ward

The most common clinical indication for HHFNC initiation was hypoxia (oxygen saturation < 92%) not responding to LF (defined as administration of oxygen of ≤4 L/min via nasal cannula) (Fig. 3).

Paediatric wards were the primary location to start HHFNC according to the majority of respondents (167/218, 76.6%). Other locations such as the emergency department were also considered an option when a ward bed was not immediately available. Six respondents representing 5 cardiology wards from 4 different regions reported using HHFNC for cardiac patients with different pathologies (pre- and post-heart surgery). Most respiratory physicians in this survey (24/26, 92%) considered their respiratory wards as HDU-acuity level therefore HHFNC became a standard therapy on these wards. HDU and PICU were the primary locations to start HHFNC therapy in 8/218 responses (3.6%).

Starting HHFNC therapy on the ward was overall a paediatric team-led decision, and similarly modification and weaning off HHFNC (paediatric consultant, respiratory consultant, registrar, senior nurse, or nurse practitioner) particularly in hospitals with no HDU compared to hospitals with dedicated HDUs {24/34 (70.3%) VS 17/47 (36.6%), 95%CI: 22.6–50.4%, P: .002}.

Relevant guidance on HHFNC was more available in hospitals with HDUs compared to hospitals with no HDU {36/47, (77%) VS 17/34 (50%), 95%CI: 5.9–45.6, P: .012} (Table 3):

**Table 3:** Responses in terms of HHFNC guidelines, options for respiratory support and application of supportive therapy on the wards

	Working in hospitals with HDU (*n* = 164, %)	Working in hospitals with no HDU (*n* = 54, %)	*p*-value
Decisions to start HHFNC by Paediatricians, n (%)	60 (36.6)	38 (70.3)	.0001
Availability of guidelines for HHFNC, n (%)	127 (77.4)	27 (50)	.0001
Proportion of patients using HHFNC on the ward, n (%)			
< 1%	21 (12.8)	14 (6)	.17
1–5%	47 (28.6)	22 (40.7)	.12
6–10%	34 (20.7)	1 (1.8)	.001
11–20%	12 (7.3)	1 (1.8)	.14
> 20%	2 (1.2)	9 (16.7)	.0001
HHFNC not used on my ward	9 (5.5)	6 (11.1)	.16
Don’t know	39 (23.7)	1 (1.8)	.0001
Available options for respiratory support on the ward (including HDU), n (%)			
Low Flow O2	164 (100)	54 (100)	N/A
HHFNC	155 (95)	47 (87)	.067
CPAP and/or BLPAP	152 (92.7)^a^	15 (27.7)^b^	.05
Established LTV	111 (67.7)^c^	0	
Supportive Therapy			
Aerosol therapy, n (%)			
MDI therapy without stopping HHFNC	13 (7.9)	4 (7.4)	NS
MDI therapy, HHFNC is temporarily stopped	12 (7.3)	4 (7.4)	NS
Nebulised therapy without stopping HHFNC	101 (61.6)	31 (57.4)	.58
Nebulised therapy, HHFNC is temporarily stopped	5 (3)	0	
I don’t know	15 (9.1)	7 (12.9)	.40
NGT insertion, n (%)			
Always	26 (15.8)	6 (11.1)	.38
Most of the times	84 (51.2)	22 (40.7)	.22
Sometimes	23 (14)	11 (20.3)	.67
Rarely/Never	7 (4)	4 (7.4)	
Feeding while on HHFNC, n (%)			
Strictly NBM	5 (3)	6 (11.1)	.018
May start NG/NJ feed	108 (69.5)	24 (46.2)	.002
May start oral feed	17 (10.3)	12 (22.2)	.02
Sedation^d^, n (%)			
Never	74 (45.1)	33 (61.1)	.04
Rarely, sometimes	65 (39.6)	10 (18.5)	.005
Most of the times	3 (1.8)	0	
Always	0	0	

^a^CPAP on the ward and BLPAP on HDU, ^b^CPAP only, ^c^HDU only, ^d^Chloral hydrate was the most commonly used sedative, *N/A* Not applicable, *NS* Not significant

**Fig. 4:**
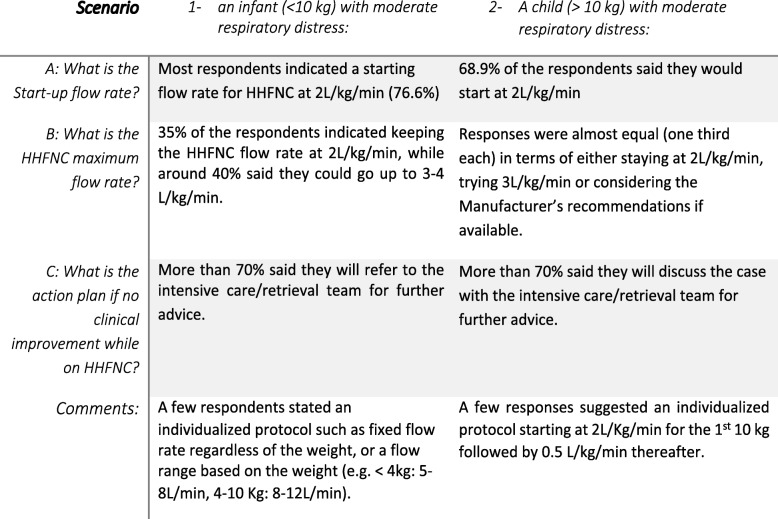
HHFNC in clinical practice on paediatric wards based on age and weight

For a better understanding of how a decision around starting and modifying HHFNC on the ward is made, we presented 2 clinical vignettes in our survey in terms of the application of HHFNC on the wards based on age and weight: findings to these scenarios are summarized in (Fig. 4). Clinical parameters by which the respondents assessed failure of HHFNC included significant work of breathing, worsening respiratory acidosis, apnoea needing stimulation, significant tachypnoea and tachycardia, deterioration on the local assessment scores (i.e. PEWS).

Weaning off HHFNC was managed variably by respondents with the majority opting to wean the FiO2 to a certain value (most commonly 0.40, indicated by 62.2% of respondents) and then gradually weaning the flow rate afterwards (75.7% of respondents).

Respondents were asked to compare between CPAP and HHFNC on the wards, nearly two-thirds of them (67.9%) said HHFNC is either the same or superior to CPAP with fewer complications (Table 4).

**Table 4:** Respondents comparison between HHFNC and CPAP on the wards

HHFNC	Efficacy (%, 95CI)	*p*-value	Complications (%, 95CI)	*P*-Value
Superior to CPAP	30.6 (24.56–37.18)	<.0001	44.3 (37.59–51.16)	<.0001
Same as CPAP	37.3 (30.86–44.09)	<.0001	27.1 (21.32–33.52)	<.0001
Inferior to CPAP	7.2 (4.15–11.48)	<.0001	3.8 (1.68–7.27)	<.0001
I don’t know	19.1 (14.11–24.96)	<.0001	22.9 (17.50–29.06)	<.0001

**Fig. 5:**
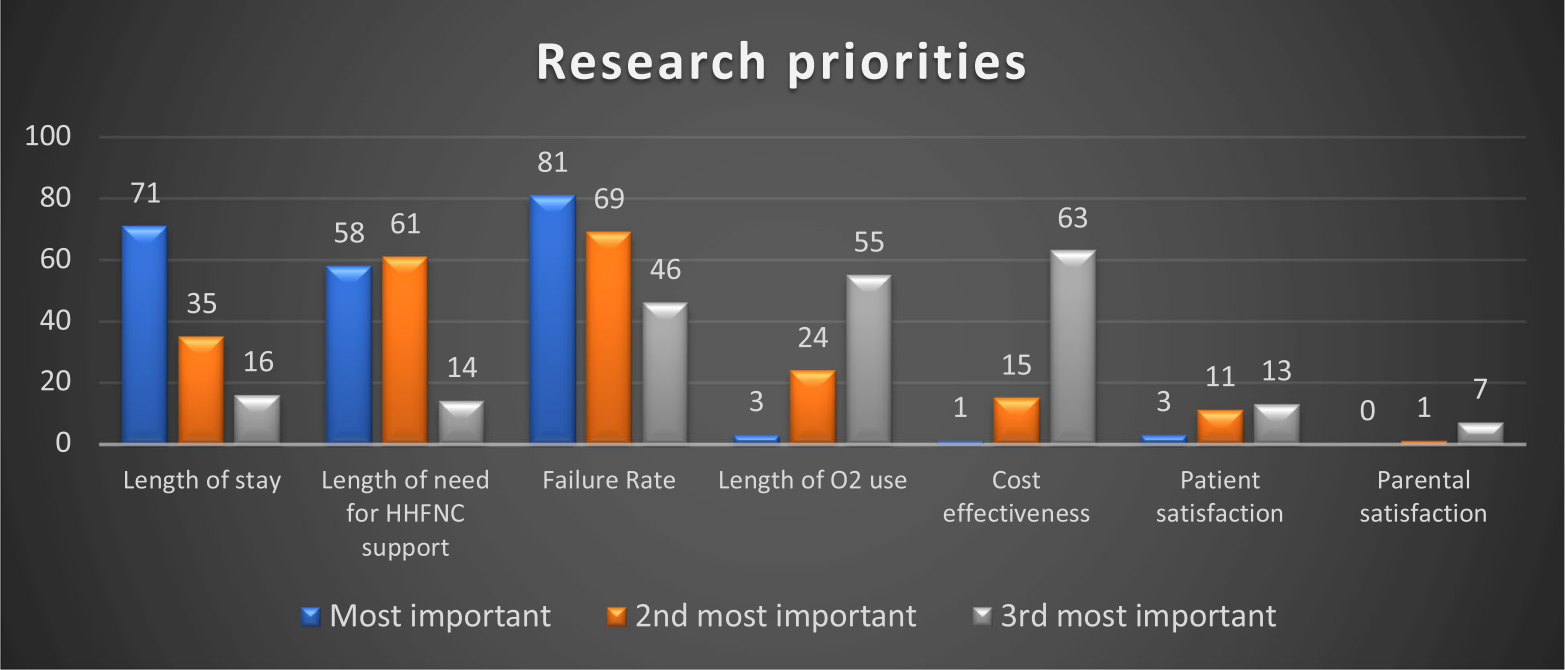
Identified Research Priorities (numbers represent responses of total 218)

Clinicians were asked to rank their three most important outcome measures on the use of HHFNC therapy in any future research (Fig. 5). HHFNC Failure rate was the first most important concern amongst the respondents followed by the length of need for HHFNC support as second most important and cost-effectiveness as third (37.1, 28, and 28.8% respectively).

Overall, 187 clinicians (85.8%) supported the idea of developing national guidance on the use of HHFNC in general paediatric practice. A small number of respondents said that such guidance is not necessary (12/218, 5.7%) and the remaining respondents were not sure if such guidelines might change current practice.

## Discussion

This study is the first and the largest national survey to review the current practice around HHFNC on the paediatric wards in terms of the number of responses, the geographical areas that have been covered across the UK and the use of HHFNC in many paediatric illnesses other than bronchiolitis, in addition to discussion about the supportive therapy while on HHFNC.

The study period was intentionally meant to span as many seasonal variations as possible to reflect practice and to minimize recall bias when HHFNC use is at its nadir in the summer.

In this survey, we noticed many areas of controversy and variation in clinical practice around HHFNC. As a non-invasive therapy, HHFNC is considered safe [18], easy to use and set-up for different age groups on the paediatric wards. When compared to LF oxygen therapy (maximum FIO2 of 40%), higher oxygen concentration could be delivered and is well tolerated [19, 20]. There is also some evidence that HHFNC in neonates and preterm babies is non-inferior to CPAP in preventing intubation and invasive ventilation [21, 22]. The reported literature is however limited when it comes to the use of HHFNC in the population beyond infancy and diseases other than bronchiolitis [23, 24]. Its role in avoiding intubation or as a step-down after extubation is yet to be tested [25]. Although the majority of the respondents have used HHFNC for patients with primary respiratory problems, others have also used it for patients with underlying cardiac (such as cardiac failure and post-cardiac surgery) and neuromuscular-type diseases (Fig. 2) [7, 26, 27].

Determining the initial HHFNC rate and the escalation strategy is still controversial as shown in this survey. While most respondents focused on 2 L/kg/min as a start-up flow, others tried a range of 3-4 L/kg/min in cases of bronchiolitis and pneumonia. Using the actual patient’s weight is a useful guide that most units have agreed on, however, it is worth mentioning though that most devices that deliver HHFNC are capped at < 2 L/kg/min for larger children with a maximum flow of 60 L/min. Majority of the surveyed units had criteria to determine HHFNC failure and an escalation strategy to an appropriate level of support demonstrated in the two case scenarios (Fig. 4).

Another area of controversy was the use of Aerosol therapy via HHFNC. The delivery of nebulized medicines is generally affected by flow, type of system used, cannula size, and type of nebulizer used [28]. Respondents didn’t specify if certain nebulizing devices were used on their wards while connected to HHFNC.

Different feeding approaches were used for patients who were on HHFNC (oral, NG, NJ, NBM). It could not be ascertained, however, if oral feeding was only allowed in the weaning phase or administered throughout therapy. In general, keeping patients NBM while on HHFNC was the least favourable method as demonstrated in this survey (Table 3).

Nearly 80% of the respondents said they either never used sedation or used it sometimes for establishing HHFNC (Table 3).

HHFNC was generally assessed by 68% of our respondents to be either equal or more effective than CPAP with fewer complications [22]. Other surveys suggested similar results when HHFNC was particularly used in the neonatal age groups [29, 30]. These results appear high considering the absence of randomised trial evidence of the effectiveness of HHFNC.

As a word of caution, this is a description of the current practice around HHFNC in the UK at the time the survey was conducted. We acknowledge that the total response rate in this survey is not large enough to make firm suggestions, however, we believe that this survey has served its purpose of highlighting the real need of further consolidated research and probably to work on developing a national guidance on the use of HHFNC on the paediatric wards similar to other countries [31].

## Conclusion

HHFNC is a rapidly evolving therapy with little data that supports its benefit in many of the paediatric diseases that have been discussed in this survey. Our survey indicates that there is growing confidence amongst paediatricians around HHFNC that may justify its increasing use in children. The key research priorities that have been identified by our respondents may help guide future studies to answer these concerns and support their clinical decisions.

## Supplementary information

**Additional file 1.** Survey questionnaire.

## Data Availability

The datasets used and/or analysed during this study are available from the corresponding author on reasonable request.

## Abbreviations

CPAP: Continuous positive airway pressure
FiO2: Fraction of inspired O2
HDU: High Dependency Unit
HHFNC: Heated Humidified High Flow Nasal Cannula
NBM: Nil Per Mouth
PICU: Paediatric Intensive Care Unit
