# Supplementary material for: A nationwide survey on the use of heated humidified high flow oxygen therapy on the paediatric wards in the UK: current practice and research priorities
Source: BMC Pediatr. 2020 Mar 6;20:109. doi: 10.1186/s12887-020-1998-1 (PMC7059285; doi:10.1186/s12887-020-1998-1)

Heated Humidified High Flow Nasal Cannula Oxygen Therapy in infants and children admitted to the paediatric wards across the UK. A national survey of current practice.

Showing 218 of 218 responses

Showing **all** responses

Showing **all** questions

Response rate: 53%

Responses merged with the following survey:

- Heated Humidified High Flow Nasal Cannula Oxygen Therapy in infants and children admitted to the paediatric wards across the UK. A national survey

1 Please indicate the region/county where your hospital is located:

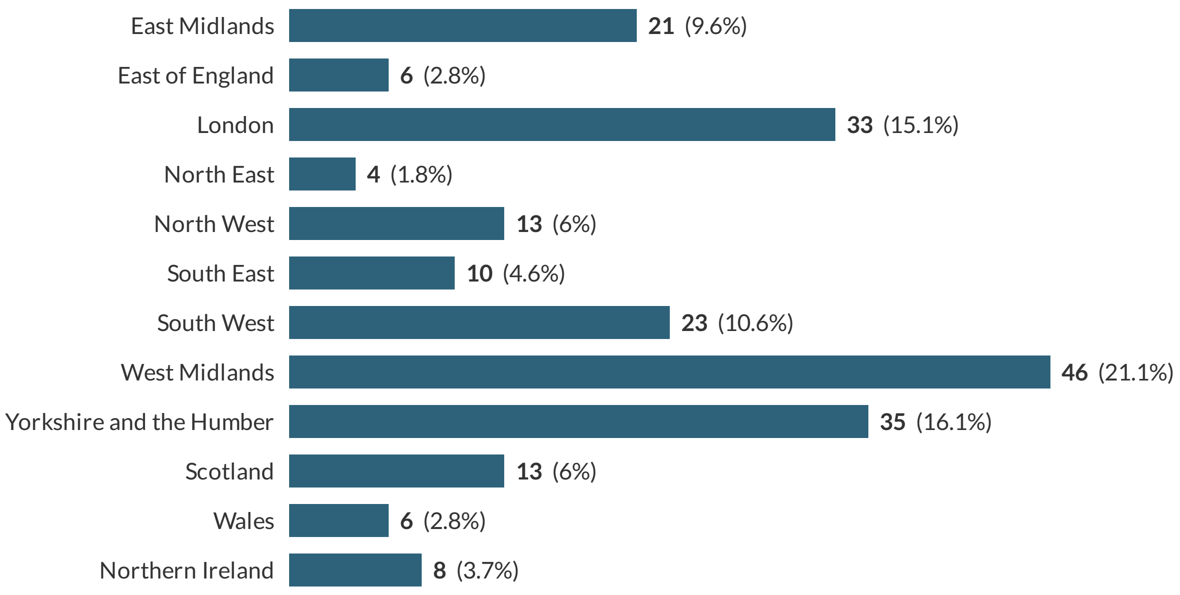

1.a Please provide the HOSPITAL NAME where your paediatric ward is located:

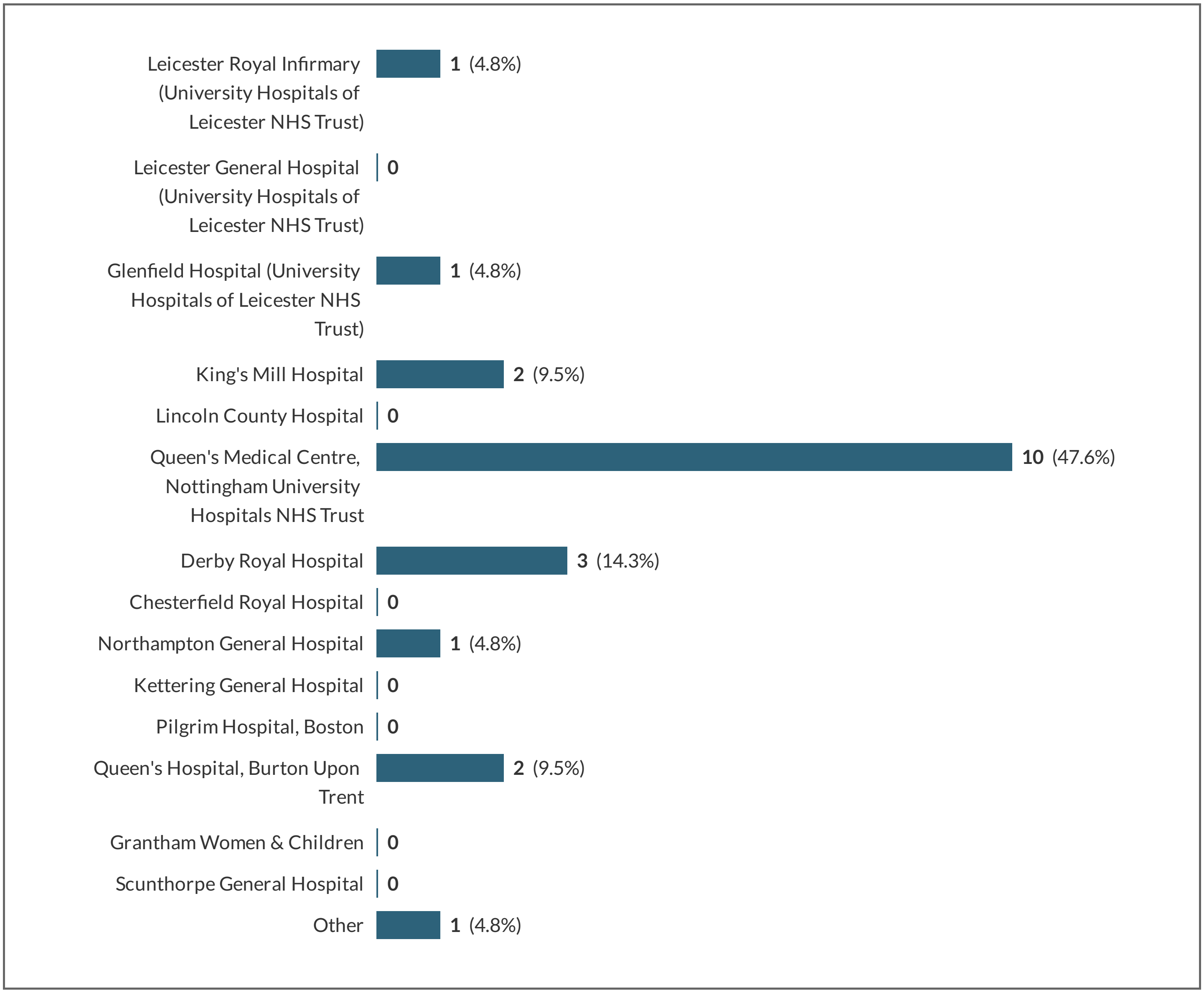

1.b Please provide the HOSPITAL NAME where your paediatric ward is located:

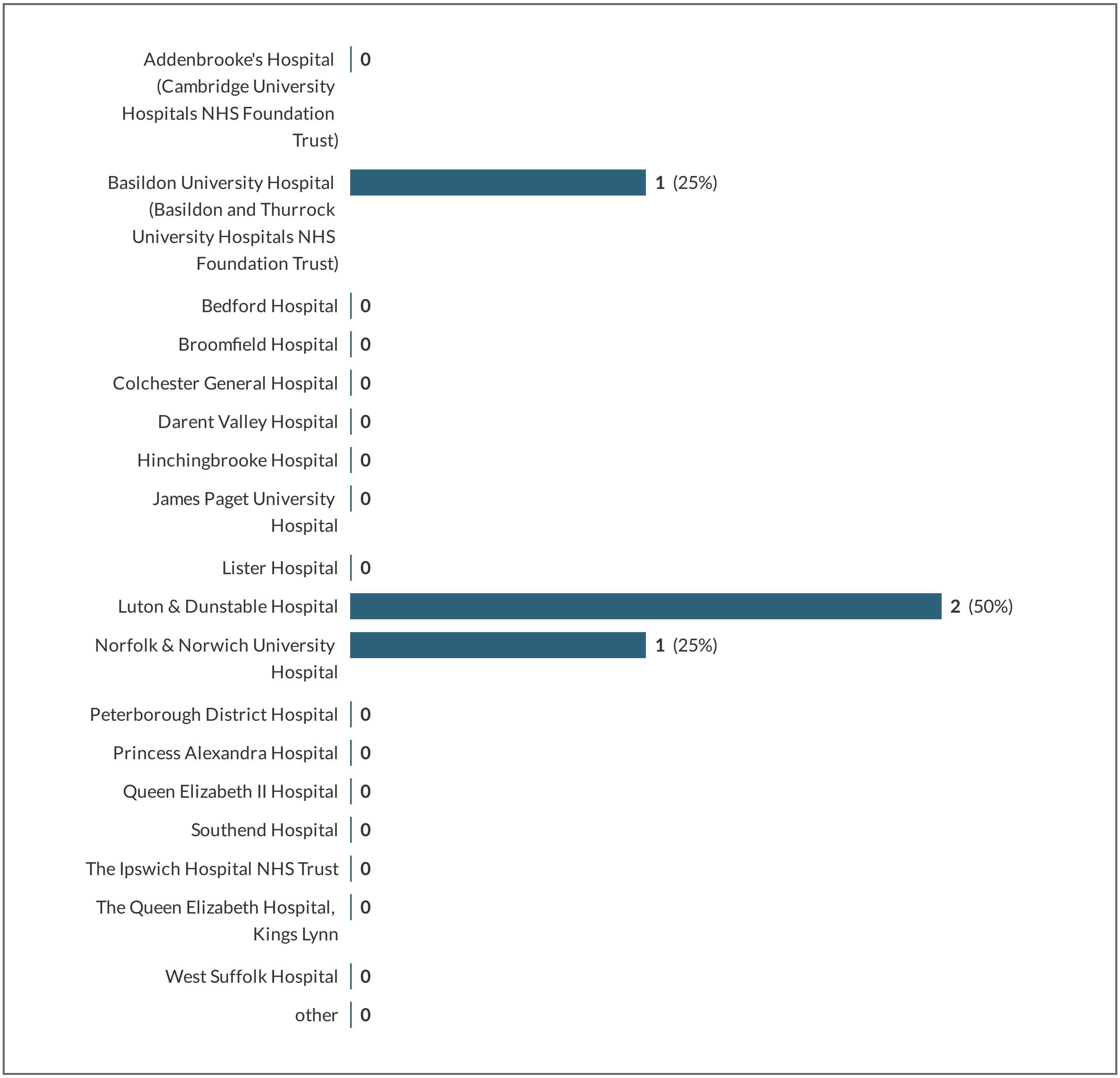

1.c Please provide the HOSPITAL NAME where your paediatric ward is located:

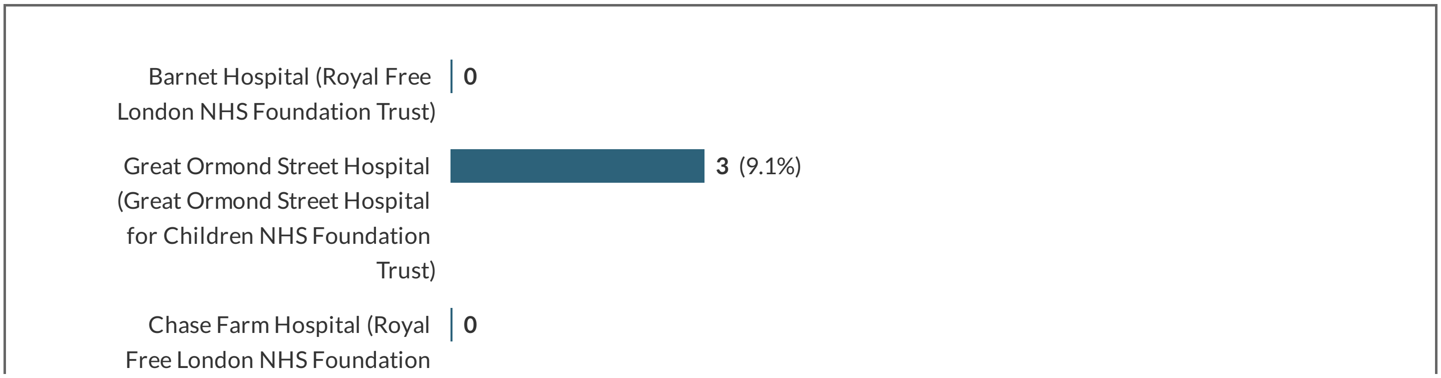

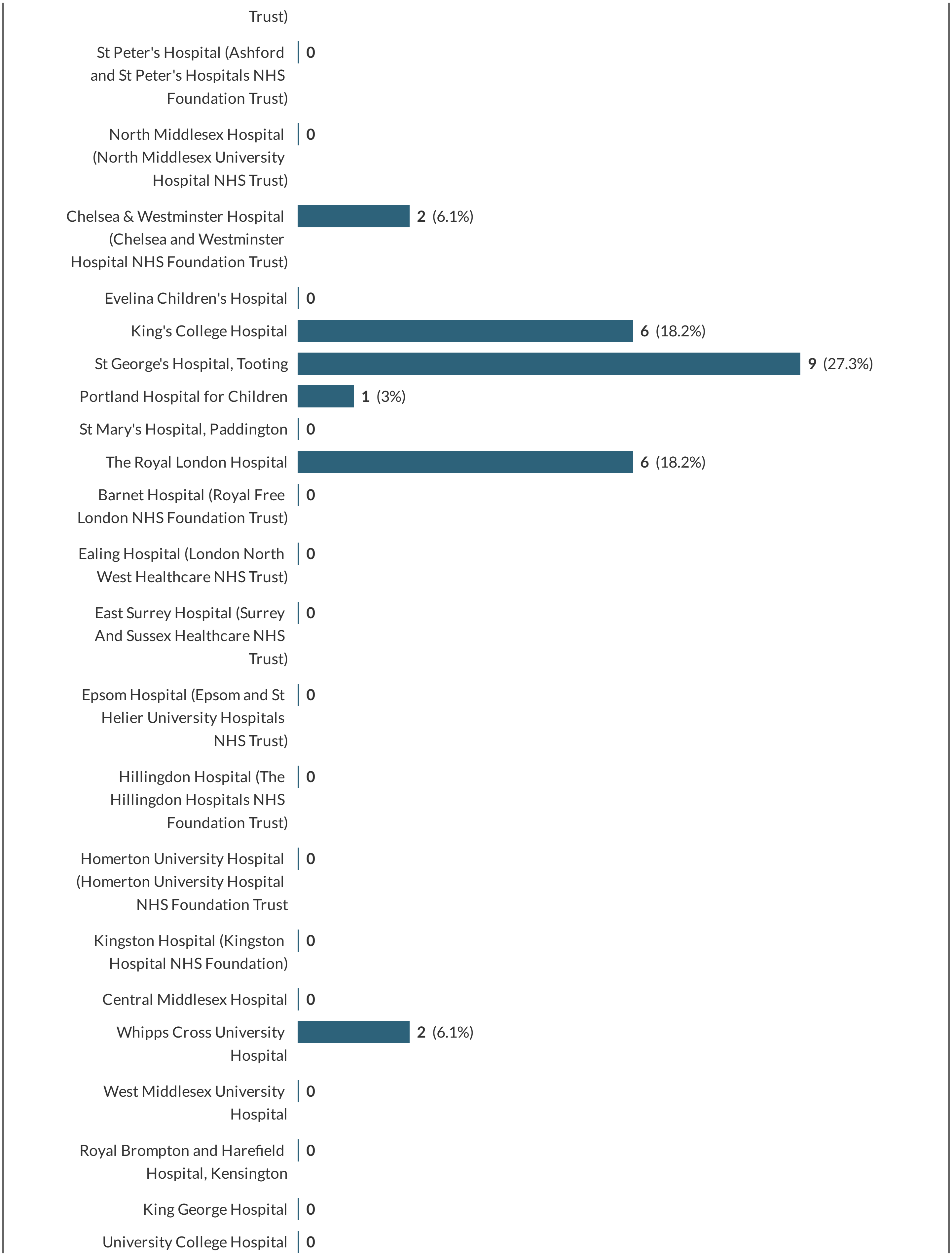
Trust)
St Peter's Hospital (Ashford and St Peter's Hospitals NHS Foundation Trust)
0
North Middlesex Hospital (North Middlesex University Hospital NHS Trust)
0
Chelsea & Westminster Hospital (Chelsea and Westminster Hospital NHS Foundation Trust)
2 (6.1%)
Evelina Children's Hospital
0
King's College Hospital
6 (18.2%)
St George's Hospital, Tooting
9 (27.3%)
Portland Hospital for Children
1 (3%)
St Mary's Hospital, Paddington
0
The Royal London Hospital
6 (18.2%)
Barnet Hospital (Royal Free London NHS Foundation Trust)
0
Ealing Hospital (London North West Healthcare NHS Trust)
0
East Surrey Hospital (Surrey And Sussex Healthcare NHS Trust)
0
Epsom Hospital (Epsom and St Helier University Hospitals NHS Trust)
0
Hillingdon Hospital (The Hillingdon Hospitals NHS Foundation Trust)
0
Homerton University Hospital (Homerton University Hospital NHS Foundation Trust
0
Kingston Hospital (Kingston Hospital NHS Foundation)
0
Central Middlesex Hospital
0
Whipps Cross University Hospital
2 (6.1%)
West Middlesex University Hospital
0
Royal Brompton and Harefield Hospital, Kensington
0
King George Hospital
0
University College Hospital
0

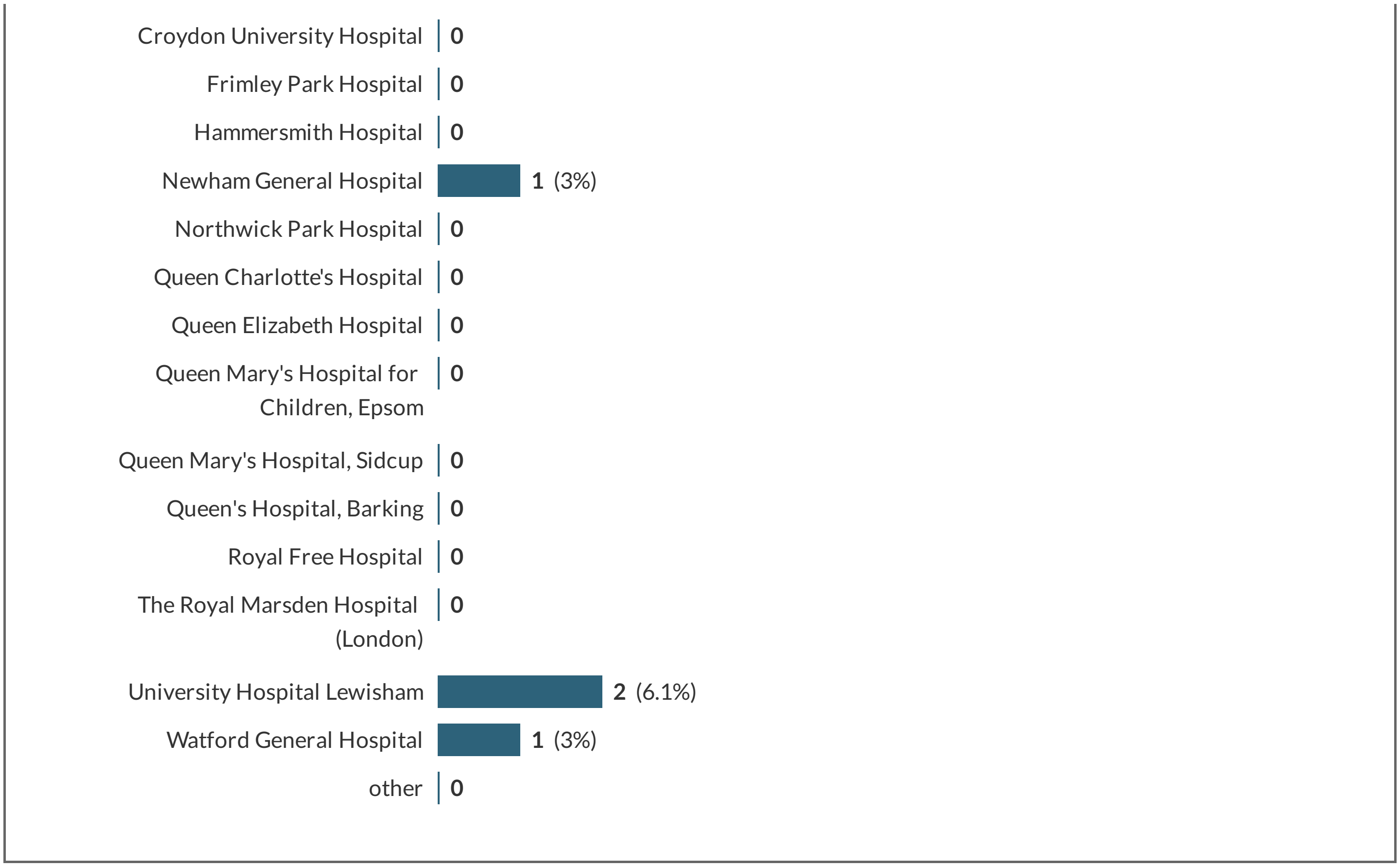

1.d Please provide the HOSPITAL NAME where your paediatric ward is located:

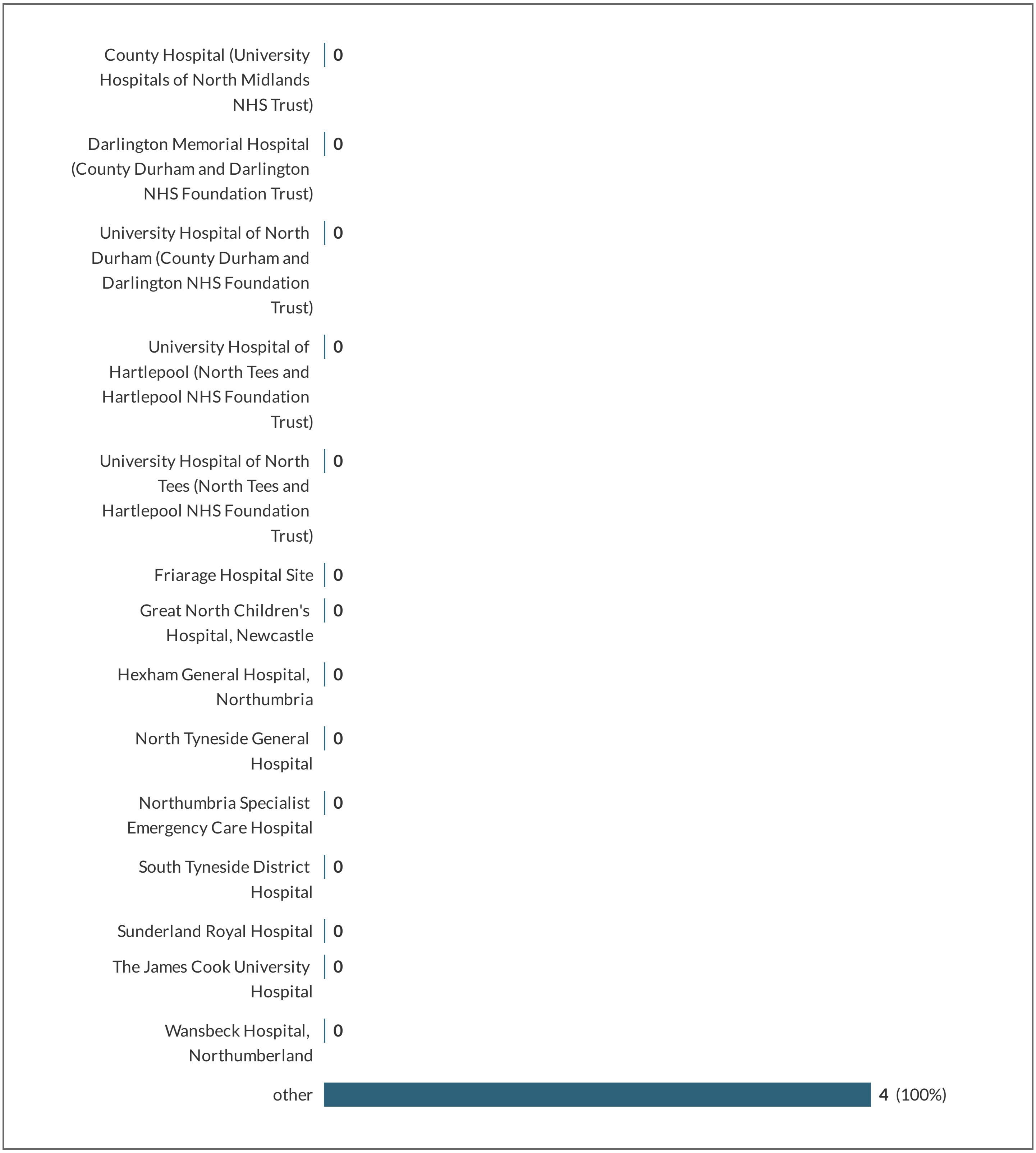

1.e Please provide the HOSPITAL NAME where your paediatric ward is located:

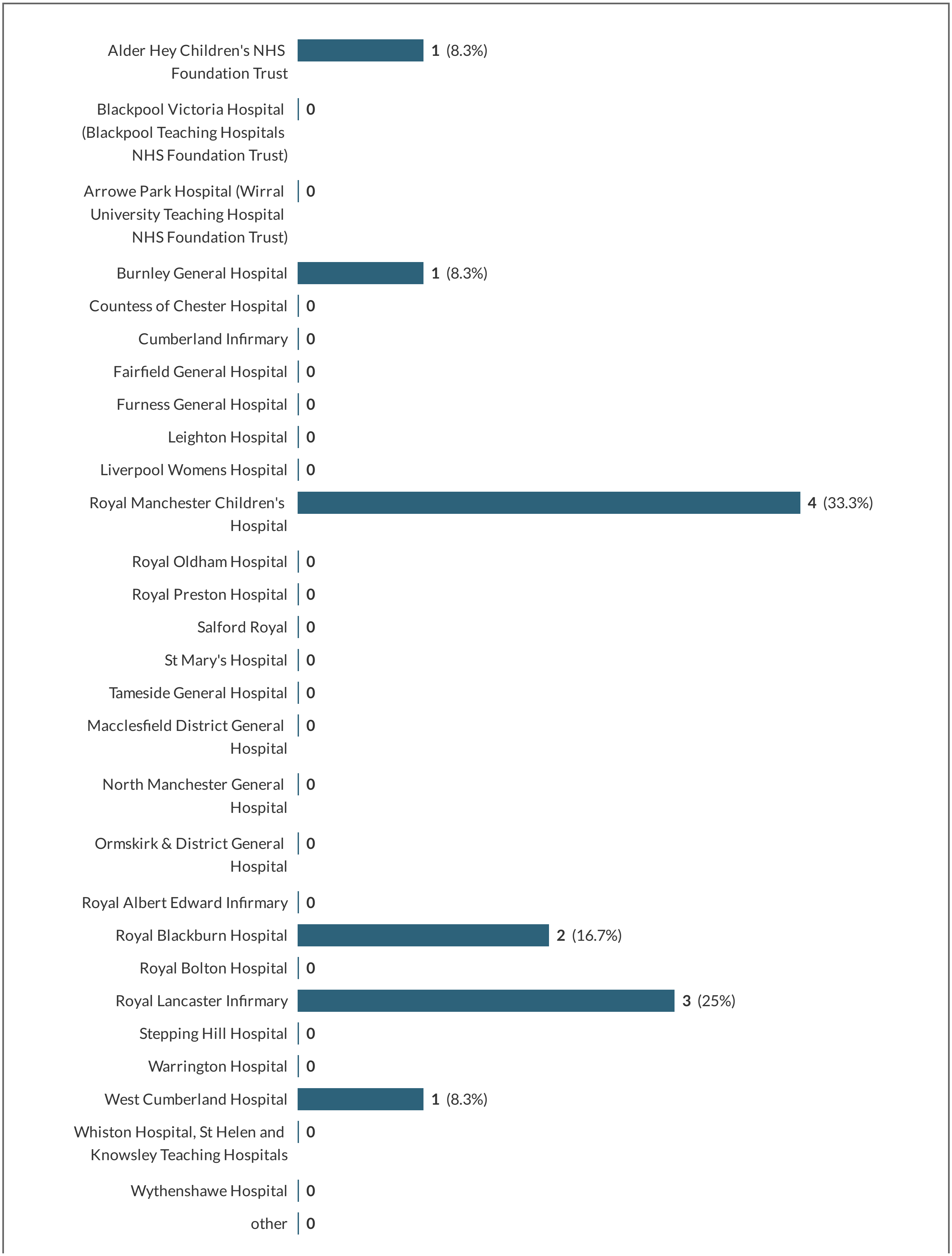

Alder Hey Children's NHS Foundation Trust
1 (8.3%)
Blackpool Victoria Hospital (Blackpool Teaching Hospitals NHS Foundation Trust)
0
Arrowe Park Hospital (Wirral University Teaching Hospital NHS Foundation Trust)
0
Burnley General Hospital
1 (8.3%)
Countess of Chester Hospital
0
Cumberland Infirmary
0
Fairfield General Hospital
0
Furness General Hospital
0
Leighton Hospital
0
Liverpool Womens Hospital
0
Royal Manchester Children's Hospital
4 (33.3%)
Royal Oldham Hospital
0
Royal Preston Hospital
0
Salford Royal
0
St Mary's Hospital
0
Tameside General Hospital
0
Macclesfield District General Hospital
0
North Manchester General Hospital
0
Ormskirk & District General Hospital
0
Royal Albert Edward Infirmary
0
Royal Blackburn Hospital
2 (16.7%)
Royal Bolton Hospital
0
Royal Lancaster Infirmary
3 (25%)
Stepping Hill Hospital
0
Warrington Hospital
0
West Cumberland Hospital
1 (8.3%)
Whiston Hospital, St Helen and Knowsley Teaching Hospitals
0
Wythenshawe Hospital
0
other
0

1.f Please provide the HOSPITAL NAME where your paediatric ward is located:

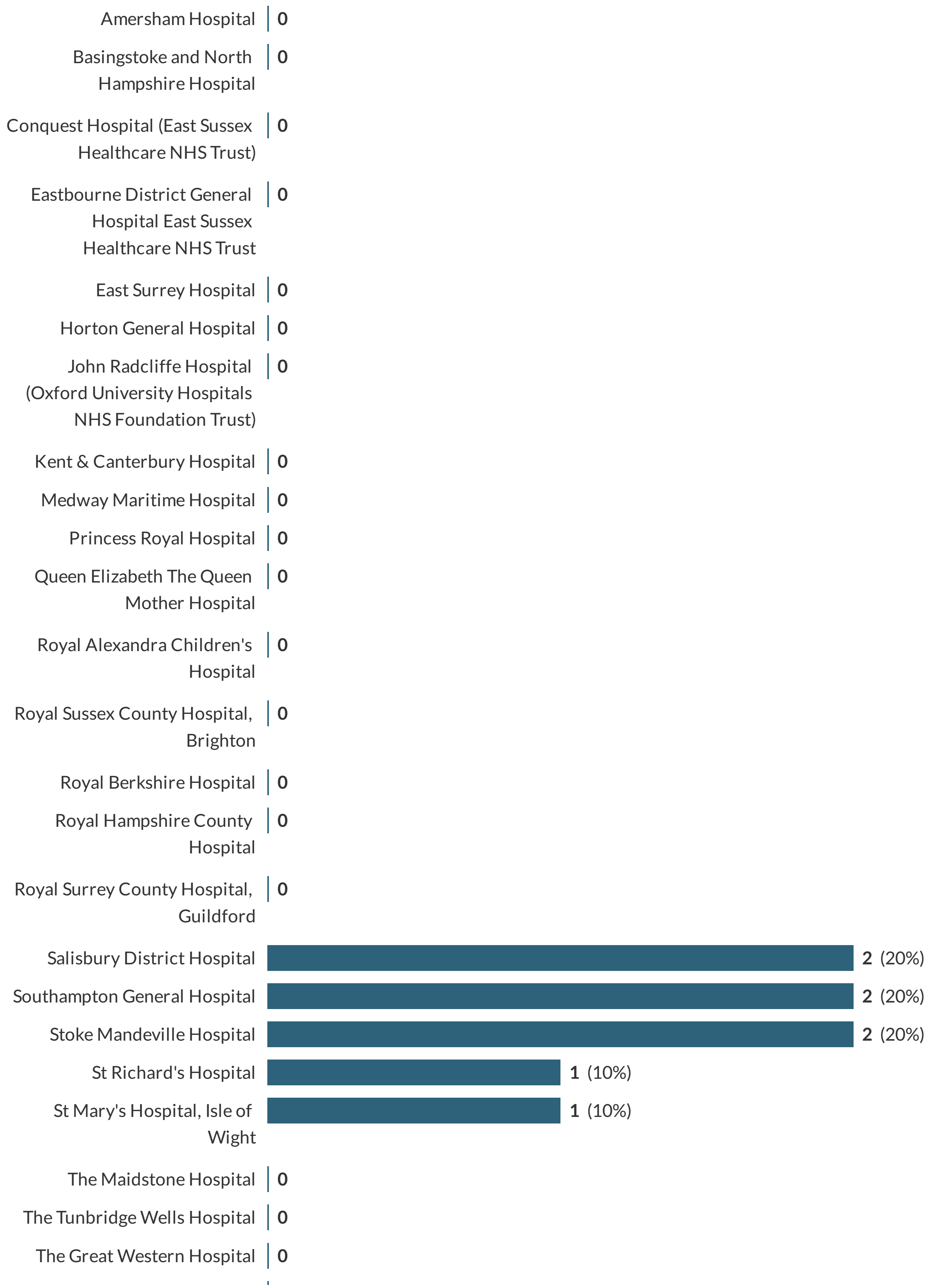

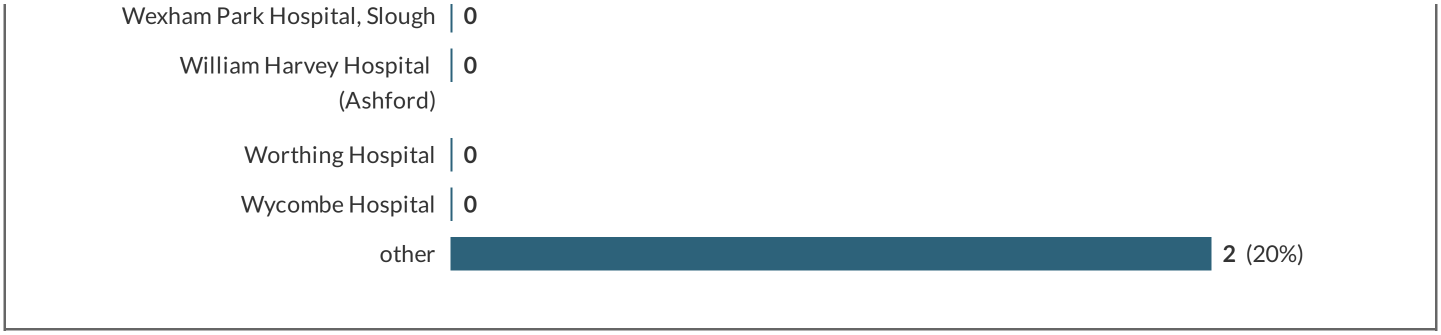

1.g Please provide the HOSPITAL NAME where your paediatric ward is located:

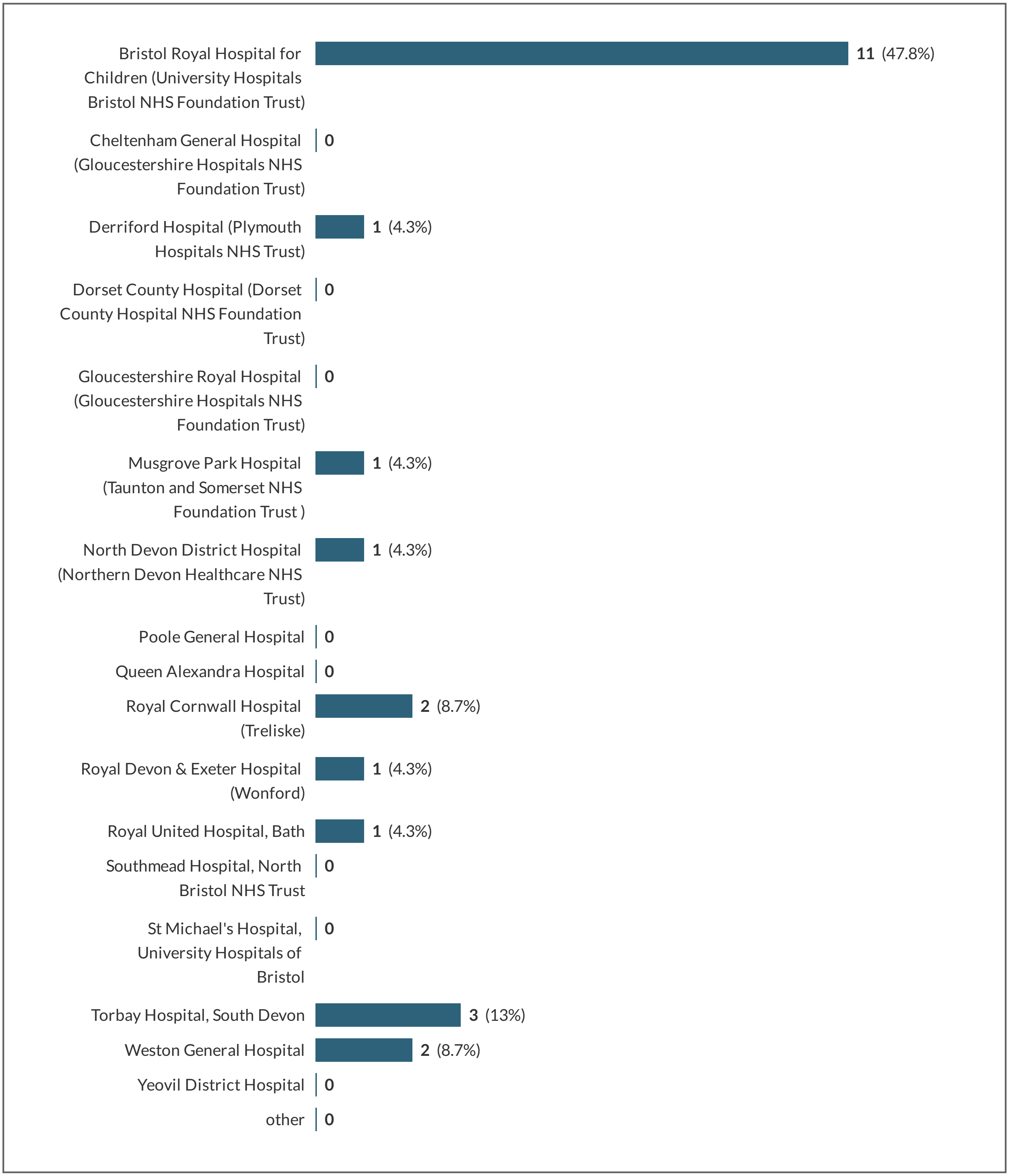

1.h Please provide the HOSPITAL NAME where your paediatric ward is located:

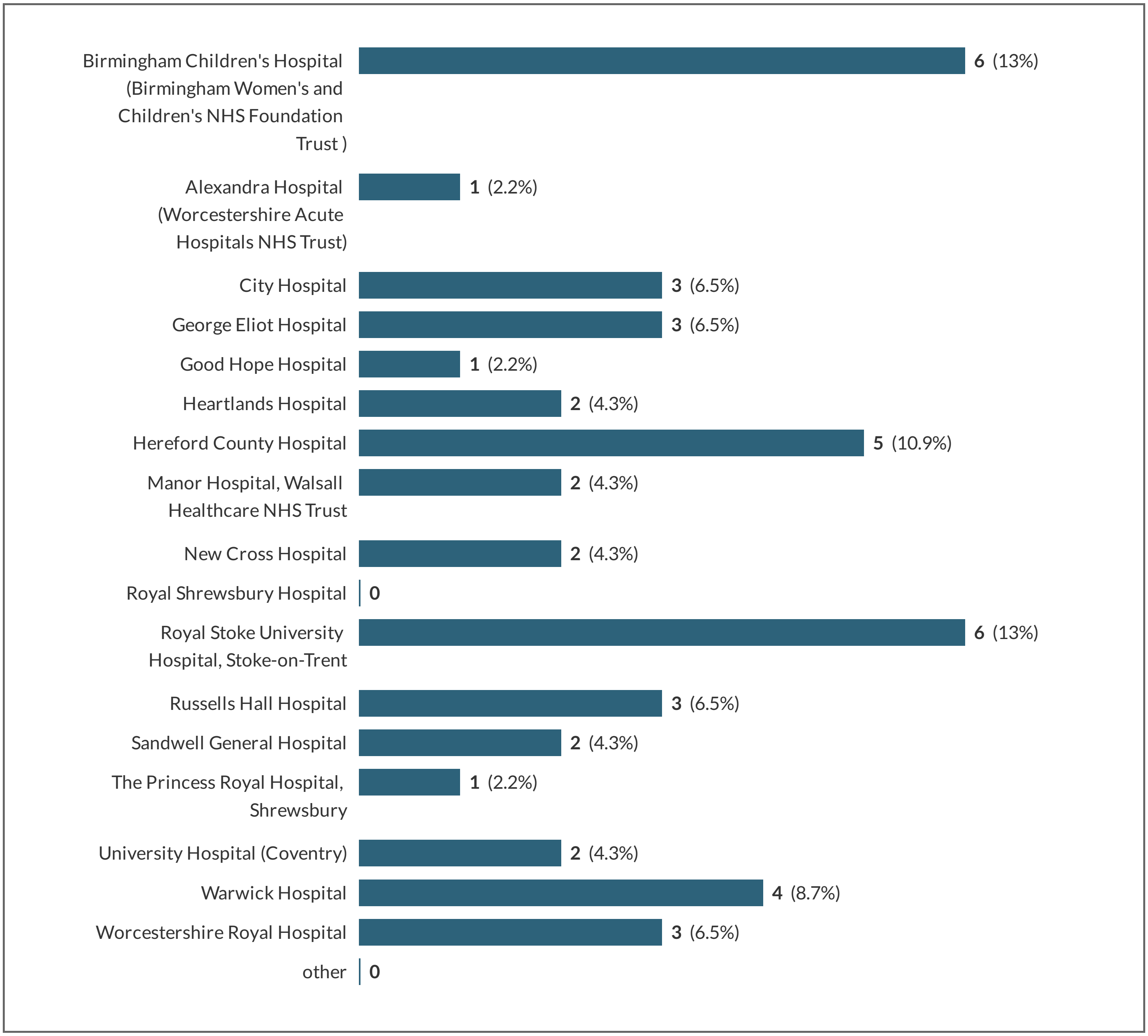

1.i Please provide the HOSPITAL NAME where your paediatric ward is located:

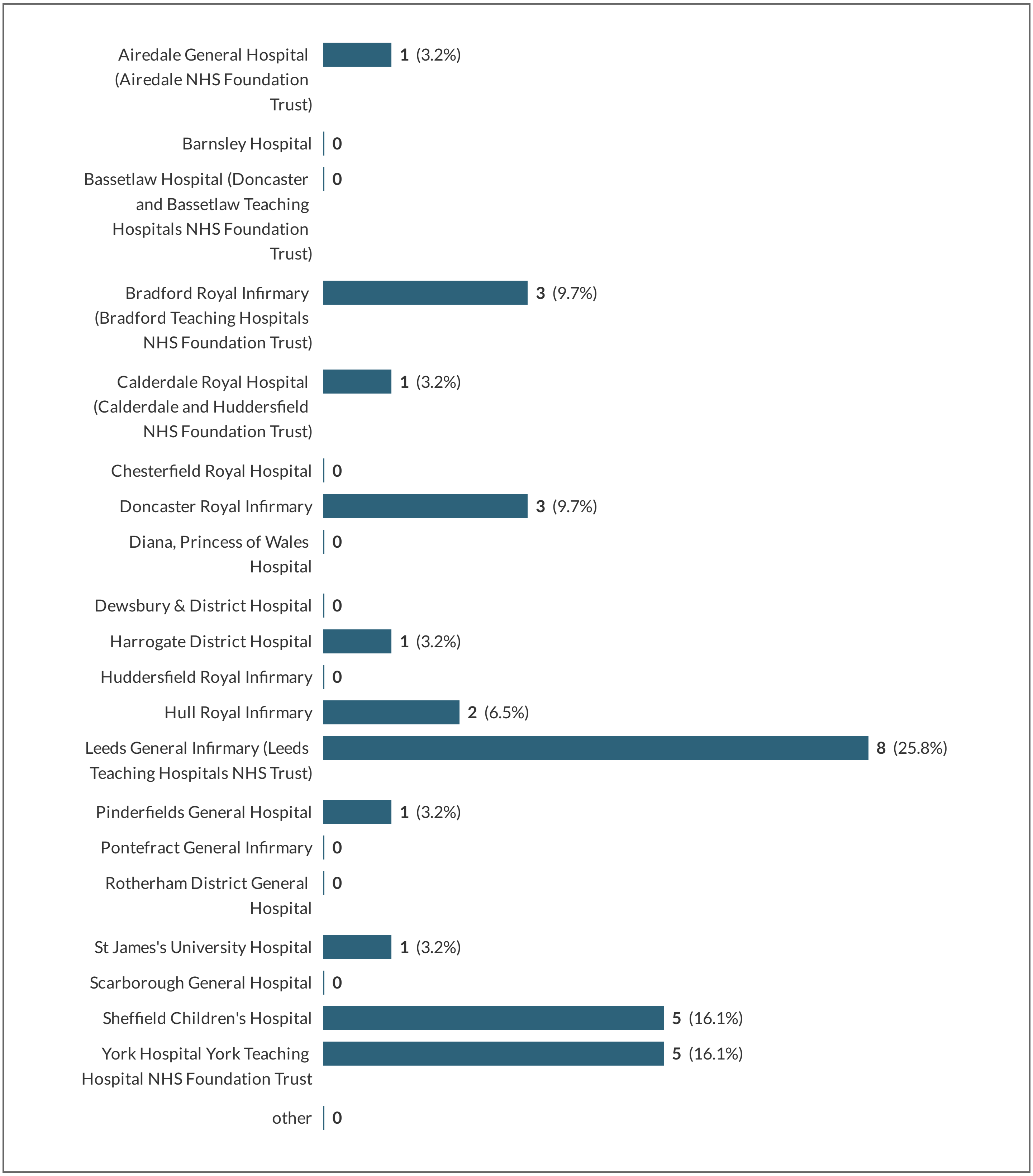

1.j Please provide the HOSPITAL NAME where your paediatric ward is located:

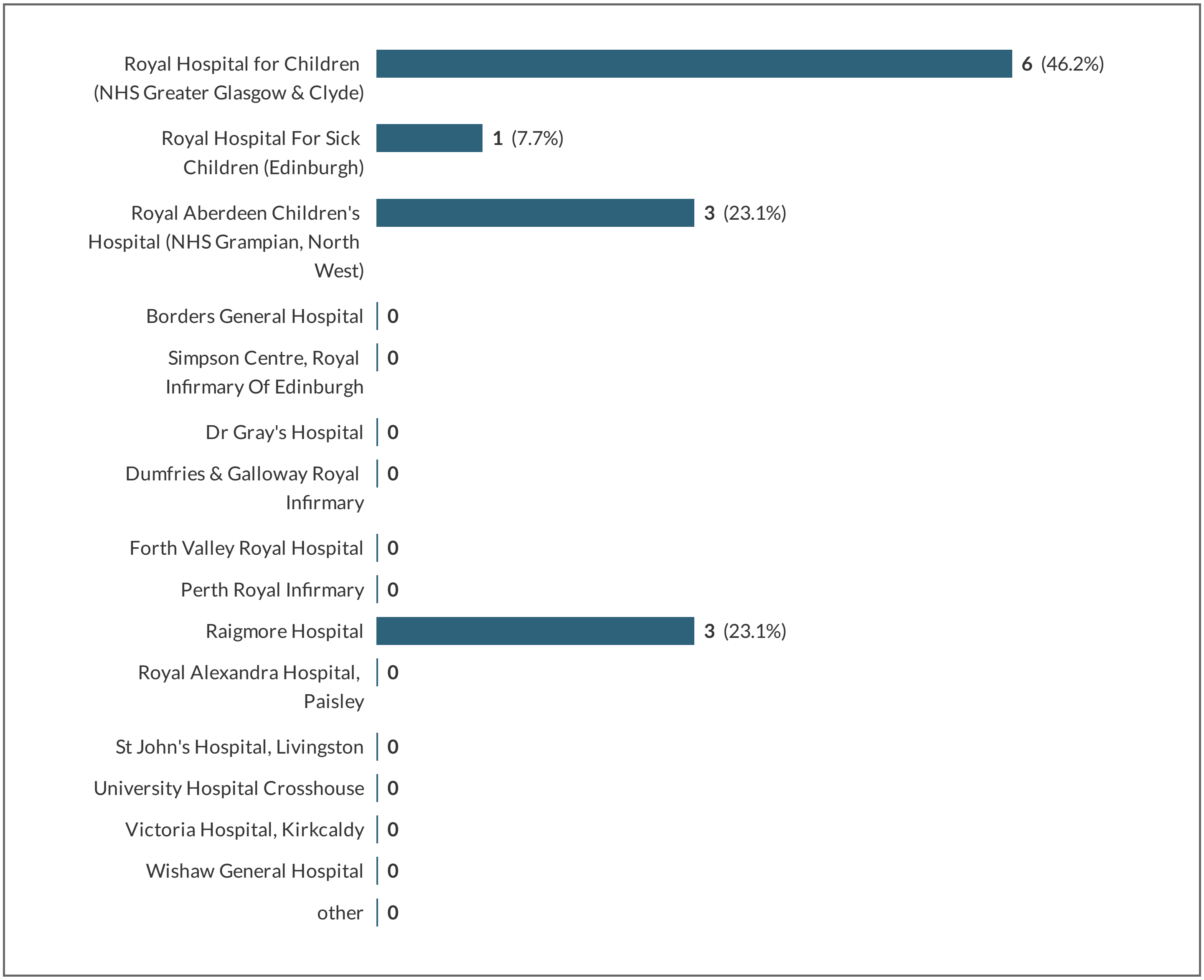

1.k Please provide the HOSPITAL NAME where your paediatric ward is located:

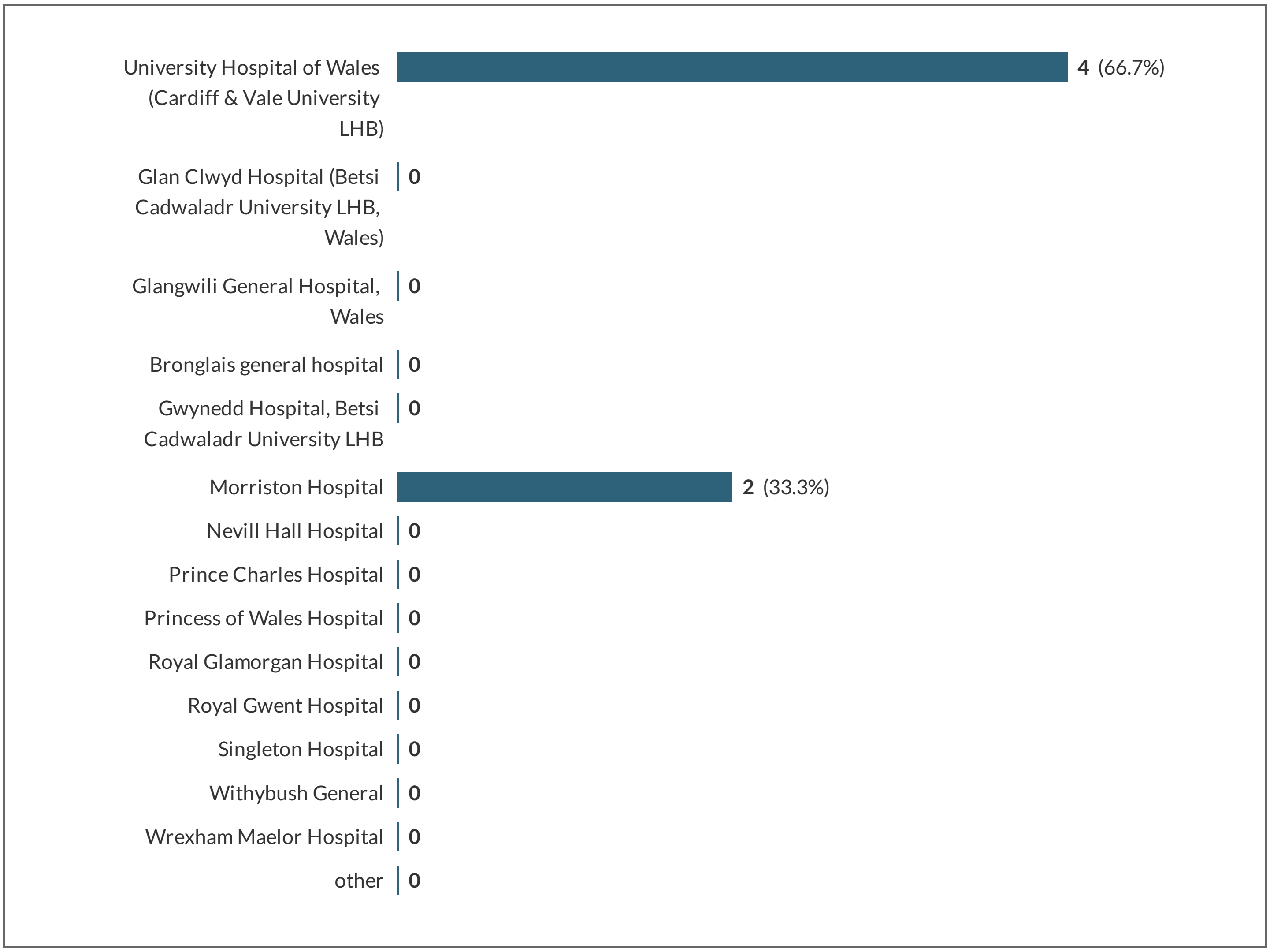

1.l Please provide the HOSPITAL NAME where your paediatric ward is located:

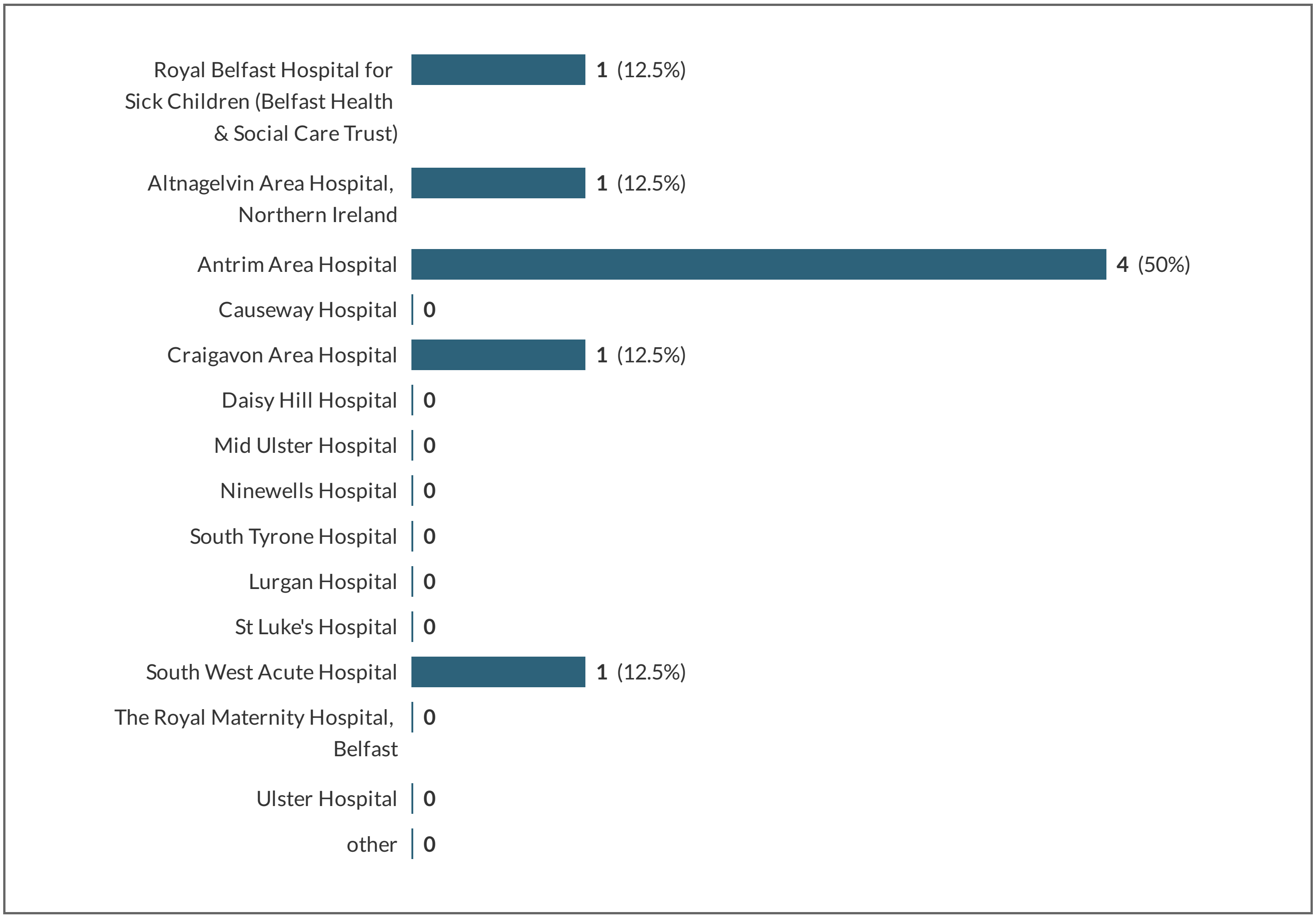

2 What is your main specialty (spending more than 50% of clinical time in this speciality) ?

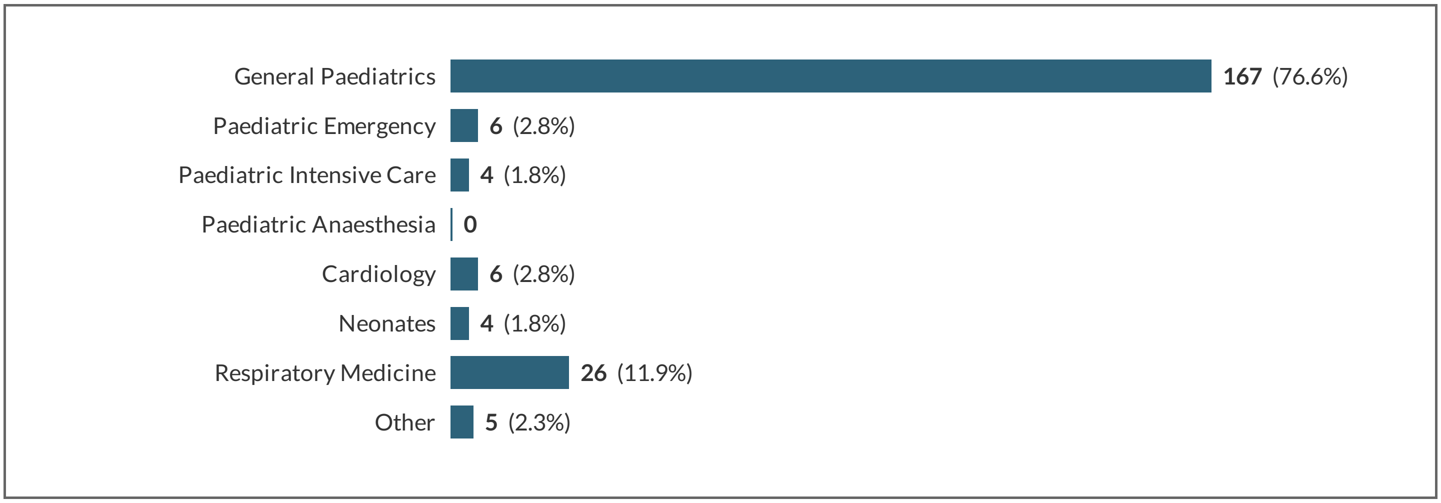

2.a If you selected Other, please specify:

Showing first 5 of 9 responses	
cardiology	369199-369190-36338389
Paediatric HDU	377609-377600-45256950
Freeman Hospital	377609-377600-45874344
Paediatric HDU	377609-377600-47769819
HDU	377609-377600-48038194

3 Years of clinical experience in the field of paediatrics (post completion of training):

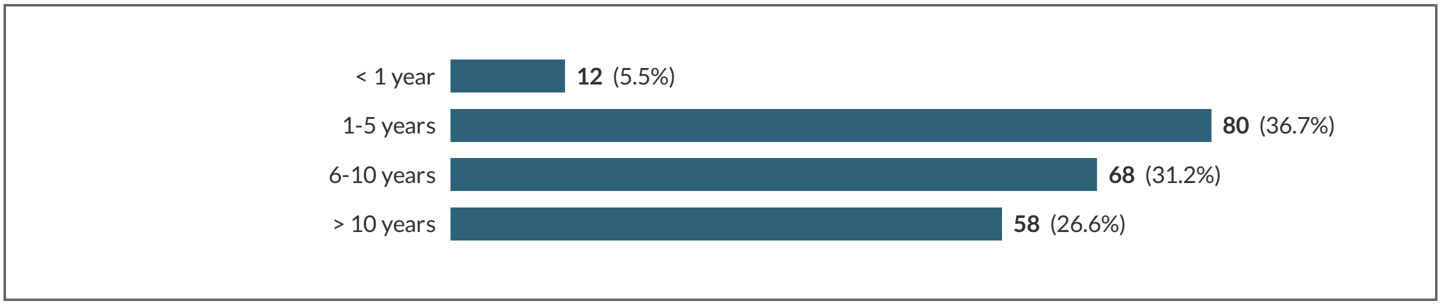

4 Please provide the maximum number of funded paediatric beds in you department: (if you don't know, insert 0)

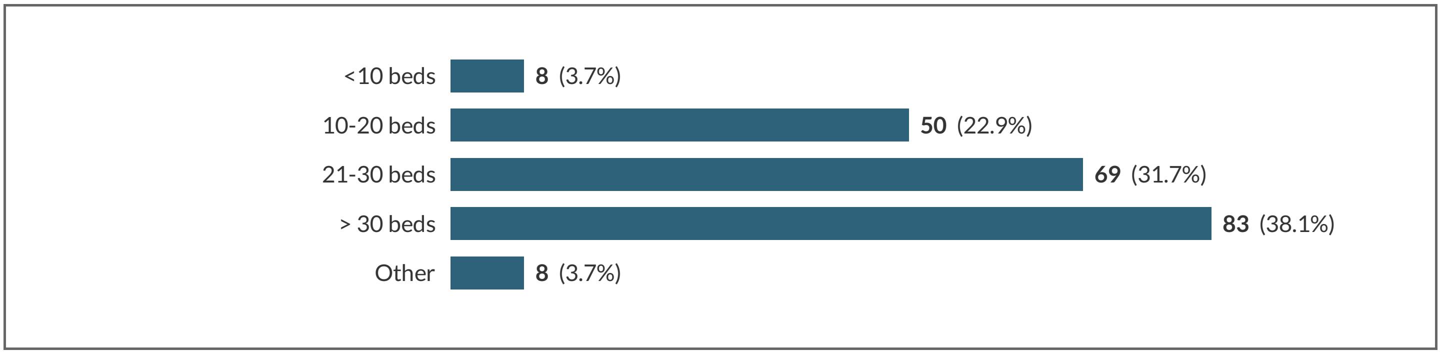

4.a If you selected Other, please specify:

Showing first 5 of 9 responses	
day case during daytime, closed overnight	377609-377600-45722958
0	377609-377600-46862936
I dont work on a specific ward but we see High Flow used in most areas of the hospital	377609-377600-48070099
3 x 6 HDU BEDS	377609-377600-48151953
0	377609-377600-48556481

5 Select the patient groups that you look after on your ward (Choose all that apply):

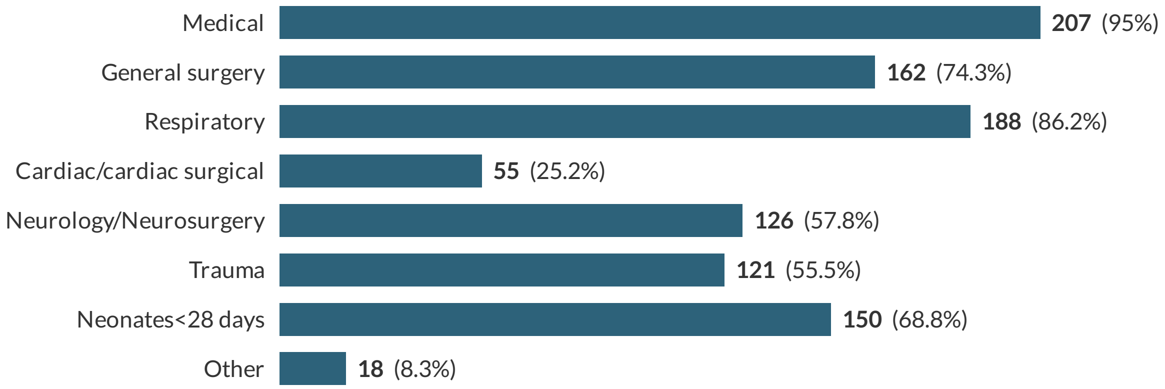

Multi answer: Percentage of respondents who selected each answer option (e.g. 100% would represent that all this question's respondents chose that option)

5.a If you selected Other, please specify:

Showing first 5 of 18 responses	
Orthopaedics , ENT, Plastics, Burns	377609-377600-39024688
Officially we don't take cardiac etc, but unofficially, given the tertiary centre is 92 miles away, we take some of everything, including babies who happen to be cardiac.	377609-377600-45201715
ENT	377609-377600-45666504
Gastro	377609-377600-45671320
All type of paediatrics patients except cardiac and liver	377609-377600-46578074

6 On average, what is the number of paediatric patients admitted to you department per year?

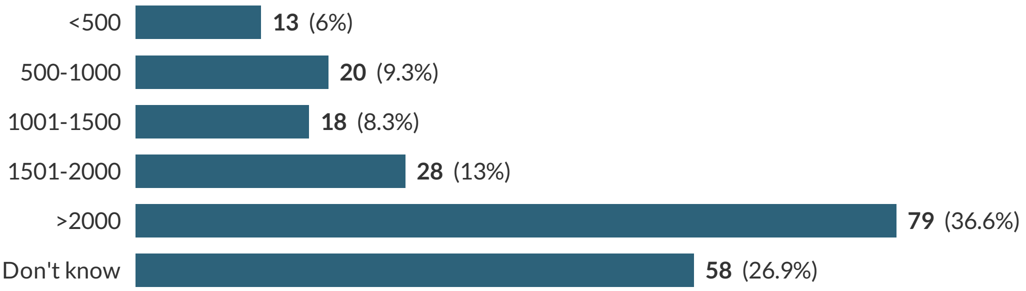

6.a On average, what is the proportion of those who require HFNC therapy?

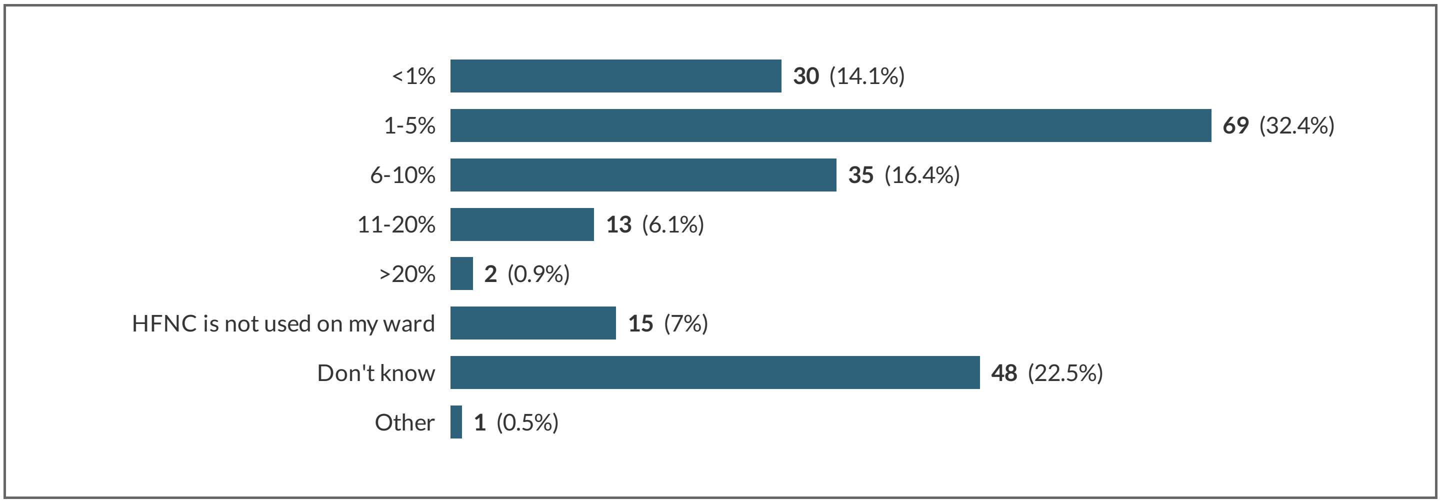

6.a.i If you selected Other, please specify:

Showing 1 response	
None	377609-377600-45722958

7 Is there a Paediatric HDU in your hospital?

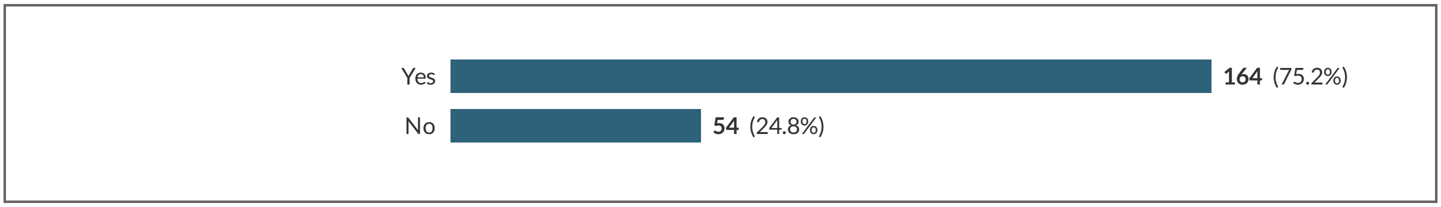

7.a If yes, this HDU is managed by:

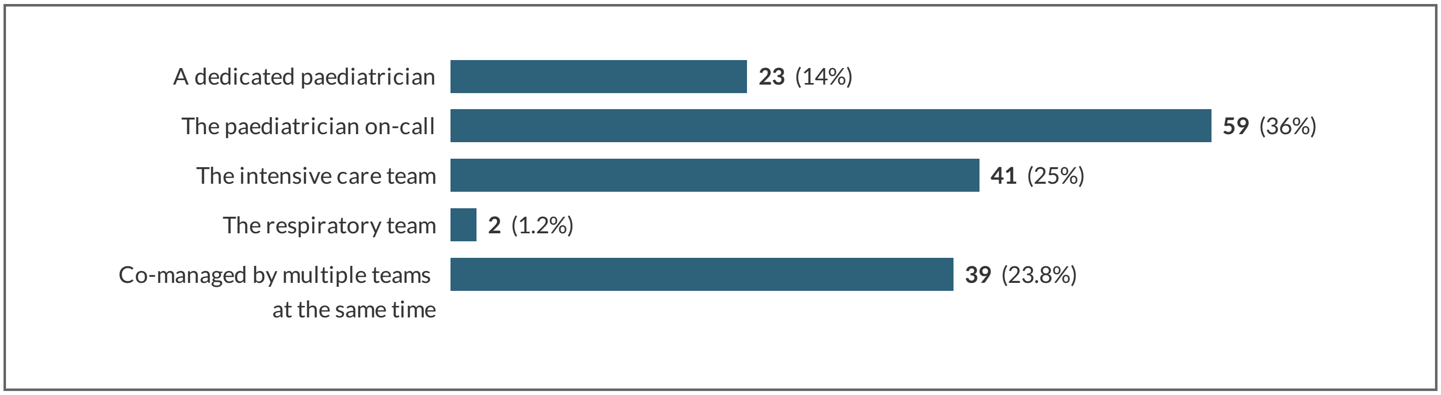

8 Available options for respiratory support on your ward including HDU (Choose all that apply): (If

you DO NOT have or use HFNC therapy in your practice, please proceed to question No: 31 on page 8).

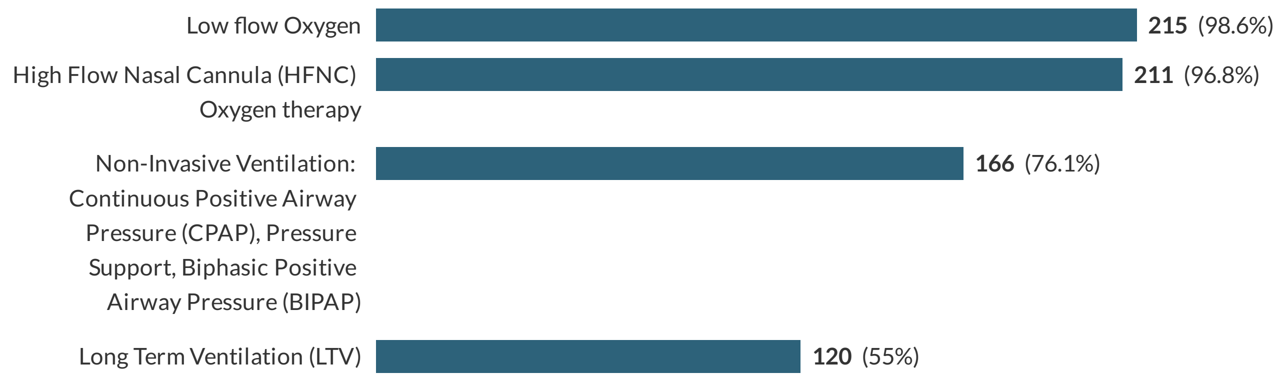

Multi answer: Percentage of respondents who selected each answer option (e.g. 100% would represent that all this question's respondents chose that option)

8.a If you do use HFNC therapy, which devices are available in your setting? (choose all that apply):

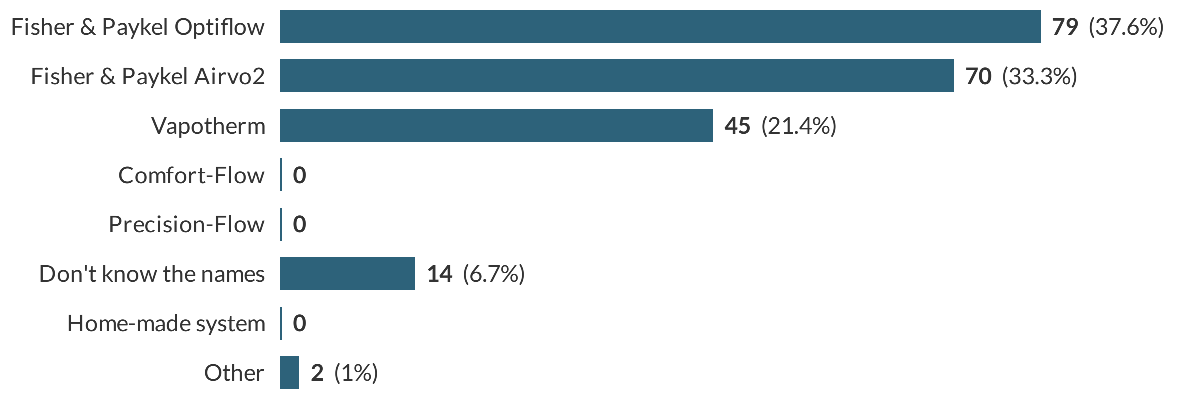

8.a.i If you selected Other, please specify:

Showing all 2 responses	
Doesn't let you choose more than 1 but have air and vapotherm	377609-377600-47623305
F+P optiflow and airvo2	377609-377600-48240470

9 For which age groups is HFNC used on your ward including HDU? (Choose all that apply):

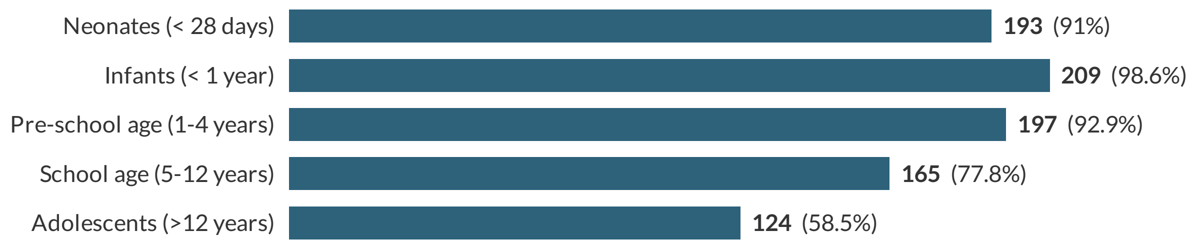

Multi answer: Percentage of respondents who selected each answer option (e.g. 100% would represent that all this question's respondents chose that option)

---

10 If a decision to use HFNC has been made, the primary location for commencing this therapy would be (tick all that apply):

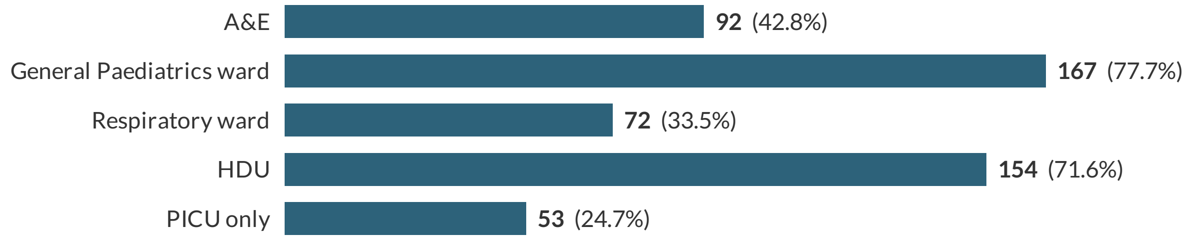

Multi answer: Percentage of respondents who selected each answer option (e.g. 100% would represent that all this question's respondents chose that option)

---

11 Who makes the decision to start HFNC therapy on your Ward including HDU? (Choose all that apply):

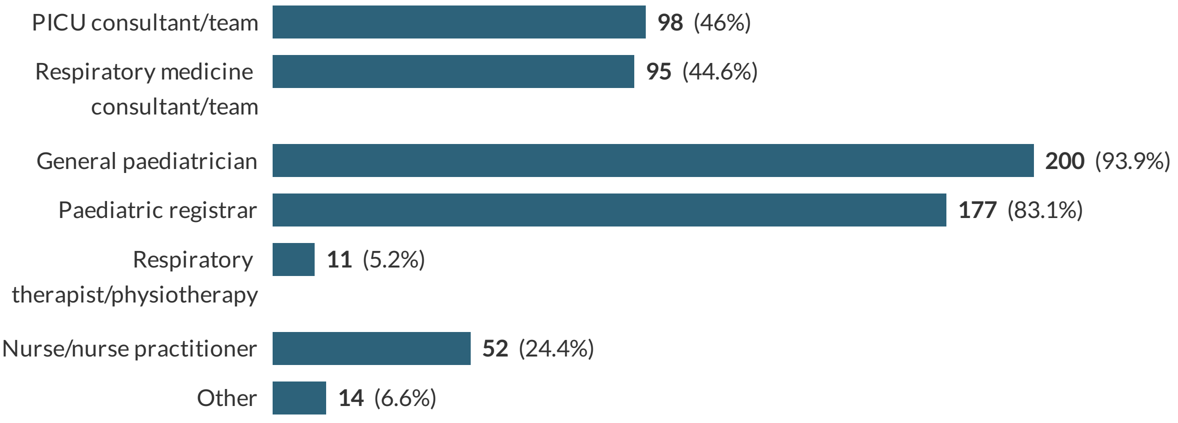

Multi answer: Percentage of respondents who selected each answer option (e.g. 100% would represent that all this question's respondents chose that option)

11.a If you selected Other, please specify:

Showing first 5 of 14 responses	
Paediatric anaethetist	377609-377600-39030543
With advice from PICU KIDS team for >1 year	377609-377600-44666197
On the ward - ARU consultant. On HDU - HDU consultant	377609-377600-45256950
Cardiology	377609-377600-45268197
Cardiology	377609-377600-45297288

12 Who makes the decision to MODIFY or WEAN HFNC settings on your ward including HDU? (Choose all that apply):

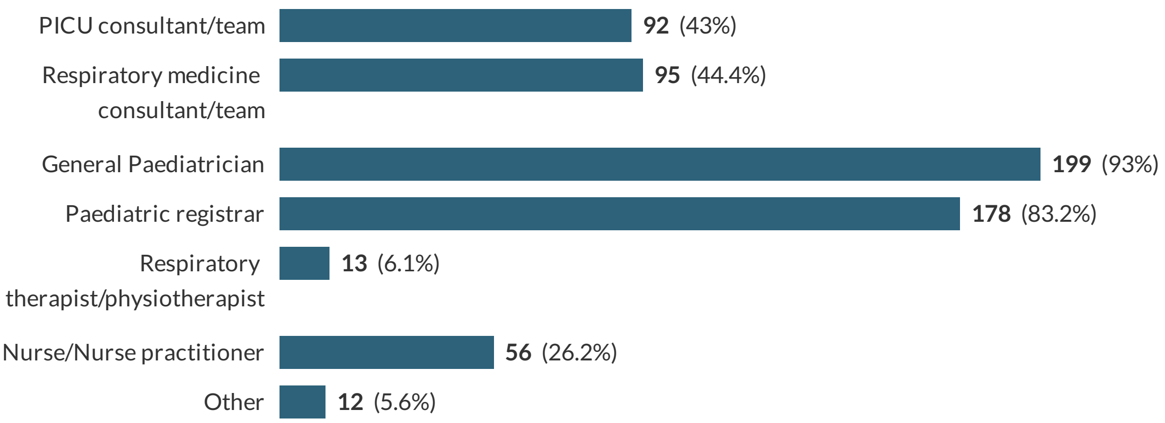

Multi answer: Percentage of respondents who selected each answer option (e.g. 100% would represent that all this question's respondents chose that option)

12.a If you selected Other, please specify:

Showing first 5 of 12 responses	
On the ward - ARU consultant. On HDU - HDU consultant	377609-377600-45256950
Cardiology	377609-377600-45268197
Cardiology	377609-377600-45297288
NICU consultant	377609-377600-45826167
PICU registrar	377609-377600-46368843

13 For which patient case is HFNC therapy used in your setting including HDU? (Choose all that

apply):

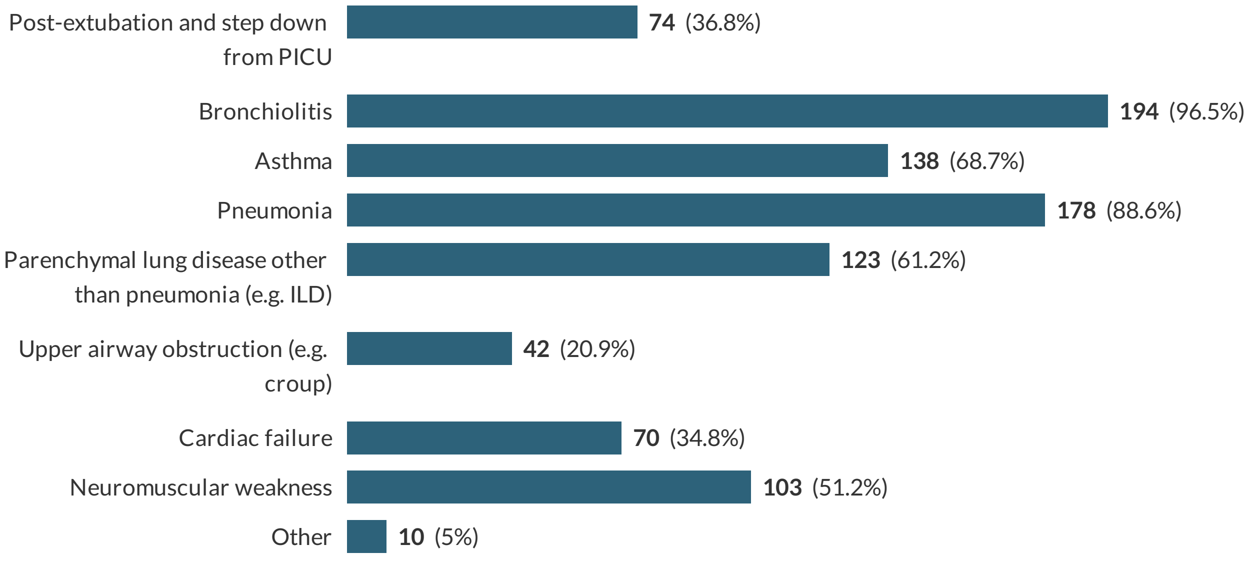

Multi answer: Percentage of respondents who selected each answer option (e.g. 100% would represent that all this question's respondents chose that option)

13.a If you selected Other, please specify:

Showing first 5 of 13 responses	
General paediatrician can start HFNC for bronchiolitis but for any other indication we need to first discuss with PICU and respiratory team	377609-377600-45192541
newborn in respiratory distress	377609-377600-47693618
Stable cardiac patients	377609-377600-47995707
It's the ITU team judgment	377609-377600-48343249
The picu team as in charge in general	377609-377600-48343496

---

14 For which clinical indications would you consider starting HFNC therapy ? (Choose all that apply):

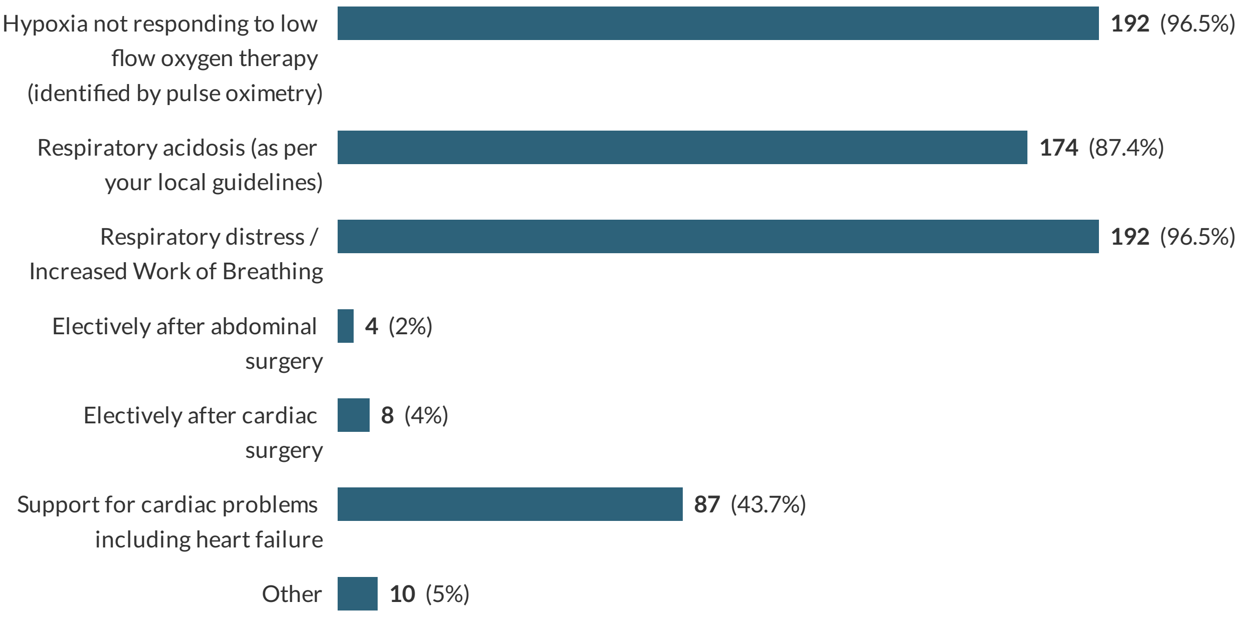

Multi answer: Percentage of respondents who selected each answer option (e.g. 100% would represent that all this question's respondents chose that option)

14.a If you selected Other, please specify:

Showing first 5 of 9 responses	
To be honest there is not standard indication. The middle grade staff think it si better than nasal prongs and NFNC is used generously in all patients admitted to HDU	377609-377600-45214559
a patient with neuromuscular disease	377609-377600-46355089
neuromuscular diseases	377609-377600-46356183
prolonged TTN in newborn	377609-377600-47693618
selectively post op and in neuromuscular/neurologypatients	377609-377600-47955614

15 Do you have a written guideline for how to start (including flow rates and nasal cannula size) and how to wean HFNC therapy in your setting?

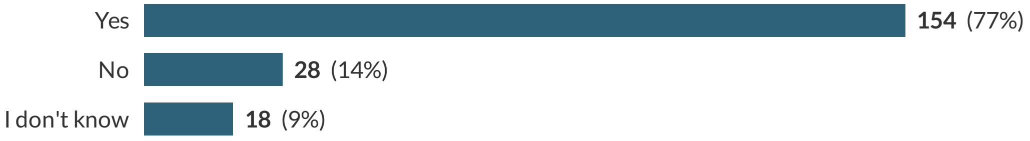

Case Study 1:

16 A previously healthy infant is admitted to your ward with moderate respiratory distress due to bronchiolitis/pneumonia. You plan to initiate HFNC as a primary therapy for the respiratory distress. What is your usual starting HFNC flow rate for an infant with moderate respiratory distress (<10 kg weight) ?

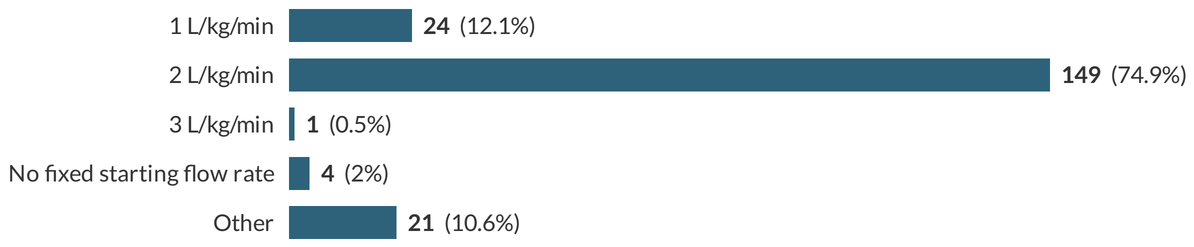

16.a If you selected Other, please specify:

Showing first 5 of 22 responses	
based on weight ranges	369199-369190-36213224
9l	369199-369190-36566089
8L/min	377609-377600-39030543
would not use HFNC as a primary therapy in bronchiolitis	377609-377600-44517386
<4kg 5-8 L/min, 4-10kg 8-12L/min	377609-377600-44666197

17 If you increase the flow, what is your maximum flow rate for this patient?

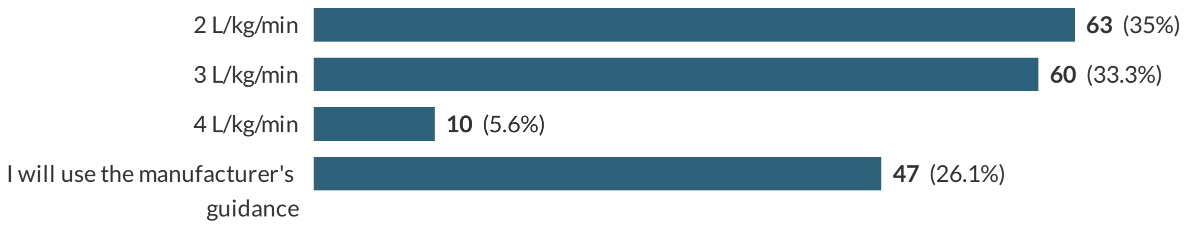

18 If HFNC at the maximum flow rate (in your practice) is not working on this patient with moderate respiratory distress, what is your next step?

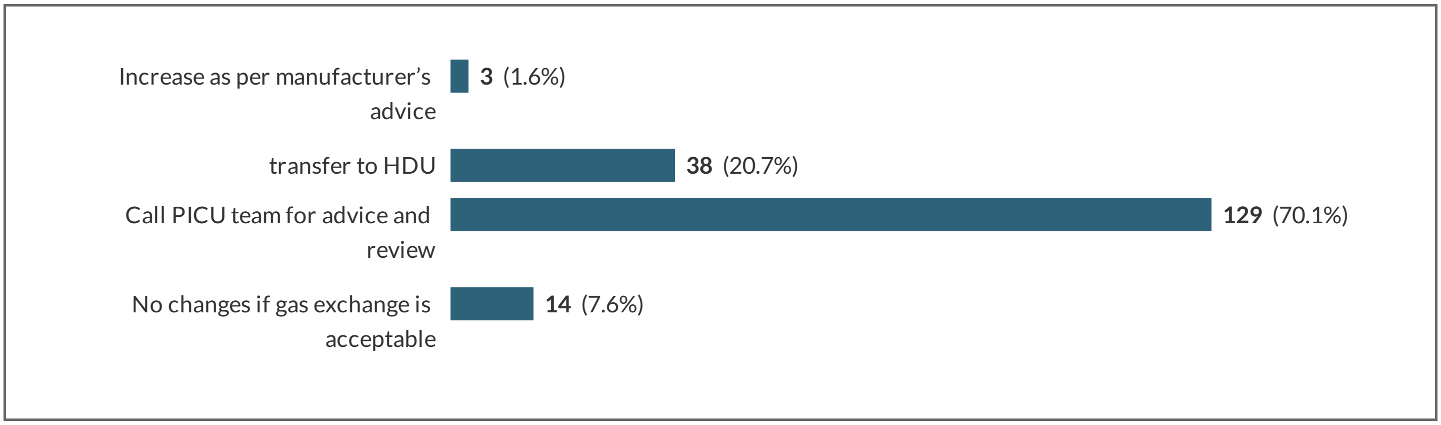

Case Study 2:

19 A previously healthy 5-year-old child is admitted to your ward with moderate respiratory distress due to pneumonia. You plan to initiate HFNC as a primary therapy for the respiratory distress. What is your usual starting HFNC flow rate for a child with moderate respiratory distress (weight > 10 kg)?

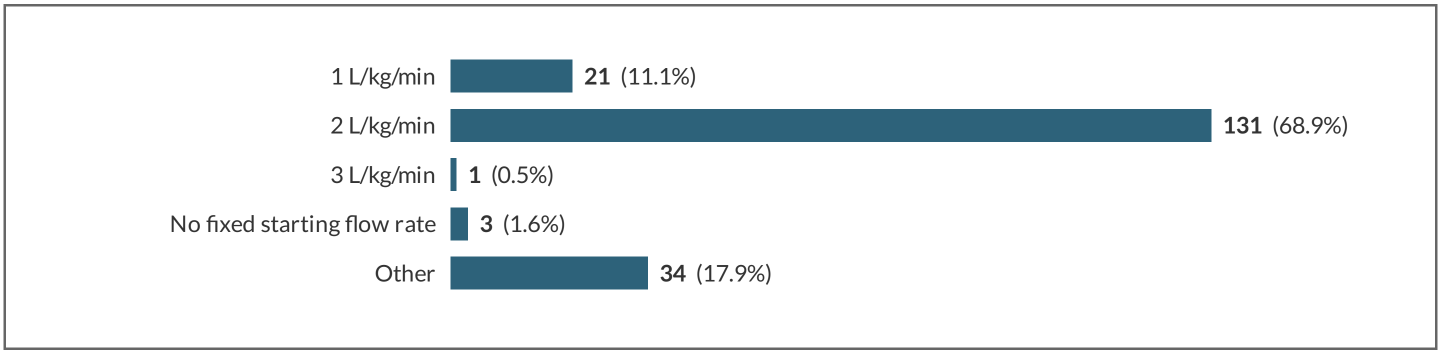

19.a If you selected Other, please specify:

Showing first 5 of 32 responses	
12L/min	377609-377600-39030543
No experience in this area - and following answers are not relevant!	377609-377600-39066761
2 L/Kg/min for 1st 10kg then 0.5 L/Kg/min	377609-377600-39117089
per kg but max flow rates according to manufacturerers	377609-377600-39813106
2L/kg for 1st 10 Kg then 1L/kg above 10 kg	377609-377600-42628053

20 If you increase the flow, what is your maximum flow rate for this patient?

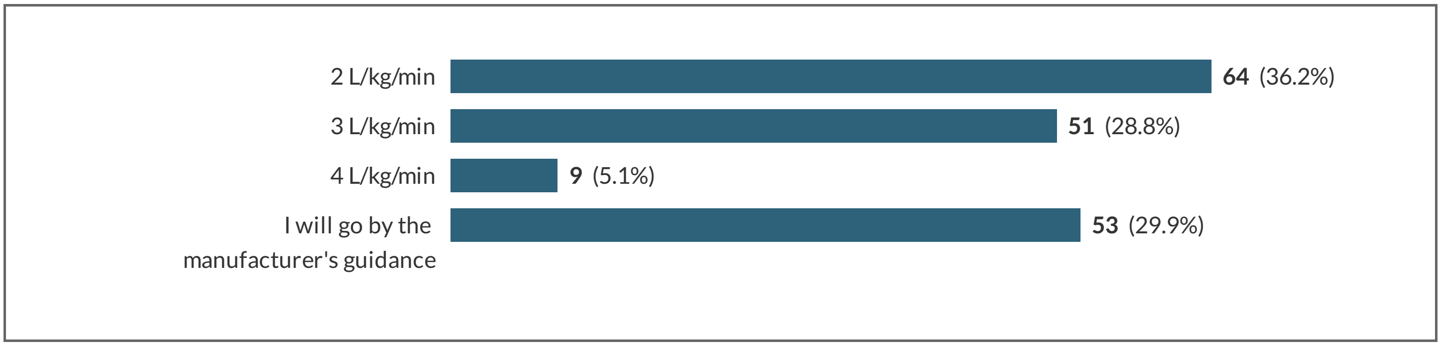

21 If HFNC at the maximum flow rate (in your practice) is not working on this patient with moderate respiratory distress, what is your next step?

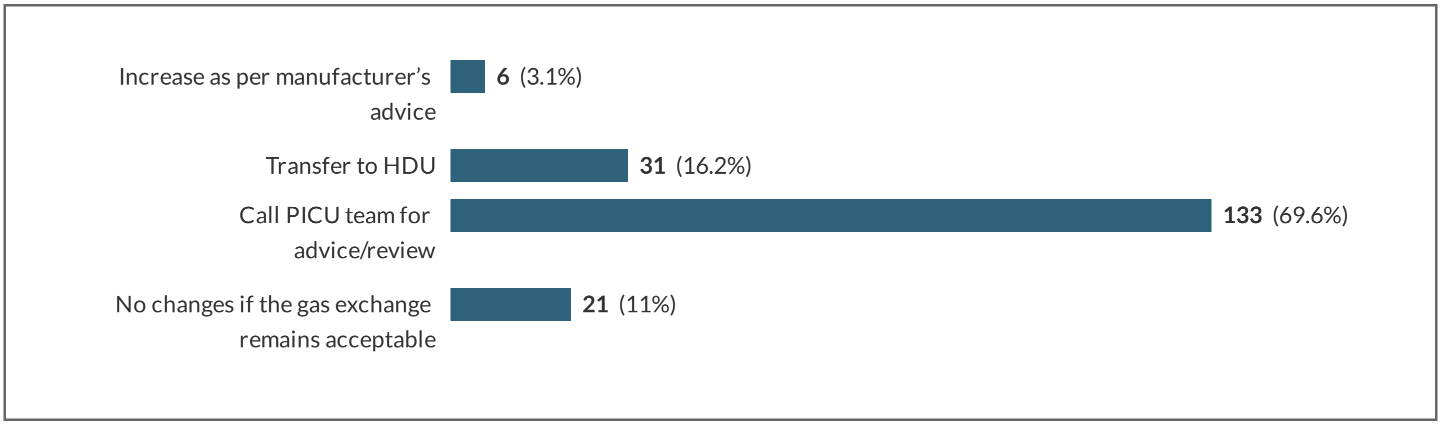

22 Which clinical markers would you mainly use to decide that HFNC therapy is not working ? (Choose all that apply):

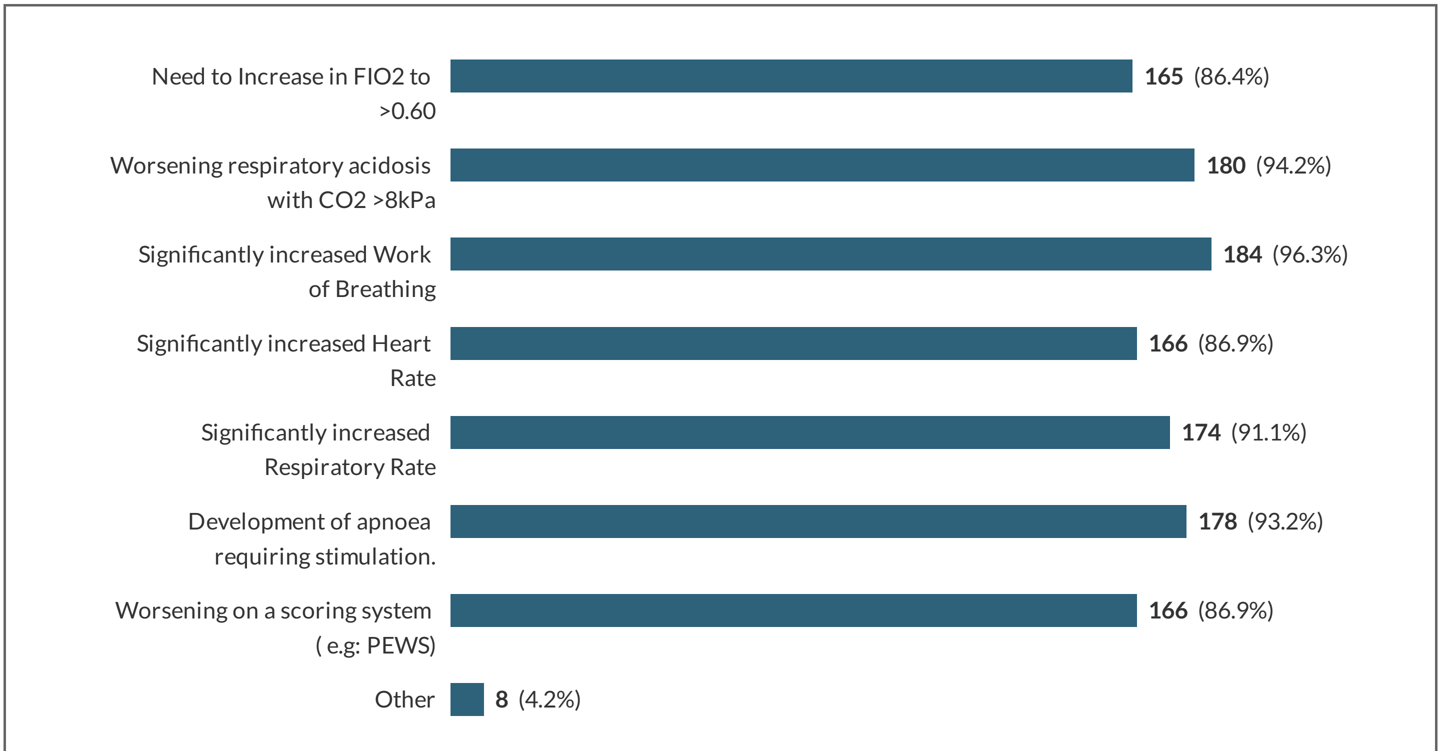

Multi answer: Percentage of respondents who selected each answer option (e.g. 100% would

represent that all this question's respondents chose that option)

22.a If you selected Other, please specify:

**Showing first 5 of 8 responses**	
Again, we are not very consistent in our approach at all. My bias is that many patietns do not need it (nasal prongs would be just as effective)	377609-377600-45214559
patient intolerance	377609-377600-46354646
in neonates need for FIO2 >0.40	377609-377600-47693618
We don't do HF on the ward	377609-377600-48651000
Not used on ward	377609-377600-48699733

23 How do you usually wean the HFNC therapy (please refer to the diagram)?

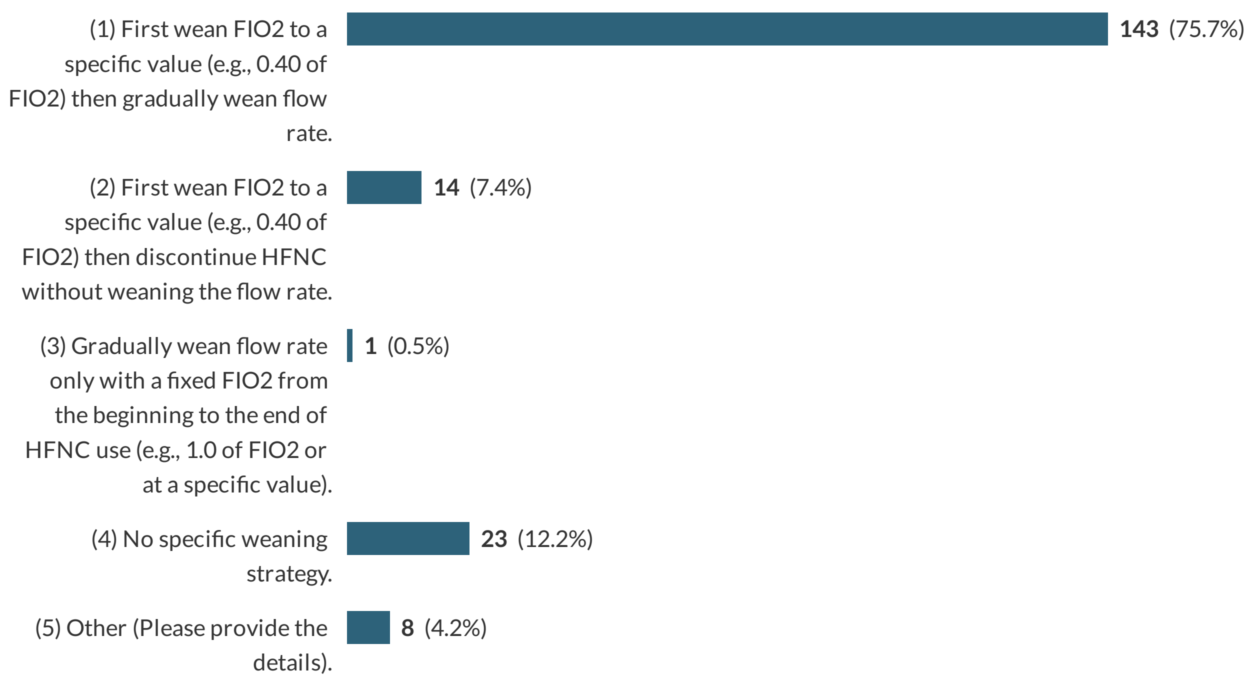

23.a If you selected Other, please specify:

Showing first 5 of 8 responses	
No specific weaning strategy as it's not used on our ward	377609-377600-48343249
Wean oxygen concentration first and then flow rate	377609-377600-48485724
Done in PICU/HDU	377609-377600-48650909
We don't do HF on the ward	377609-377600-48651000
Only on PICU. I do not wean it!	377609-377600-48699733

23.b If you choose (1), at what FIO2 do you generally start weaning the HFNC flow rate ?

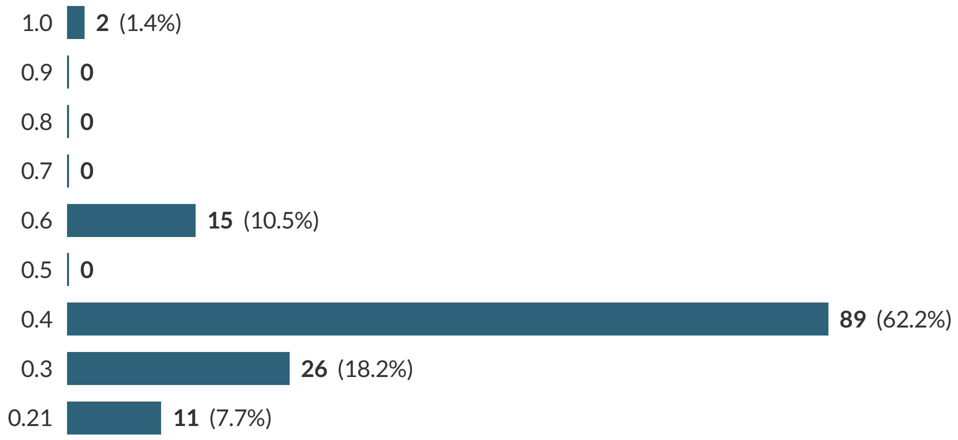

23.c If you choose (2), at what FIO2 do you generally discontinue the HFNC flow rate?

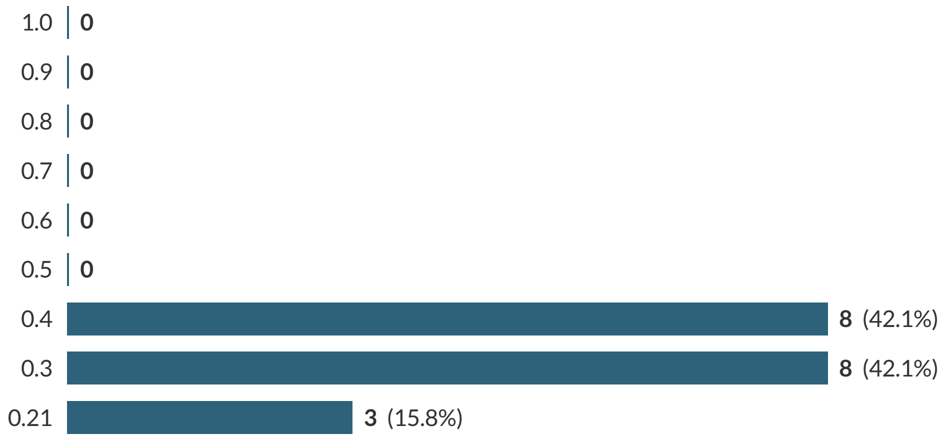

24 How do you deliver a bronchodilator when a patient is on HFNC therapy ? (Choose all that apply):

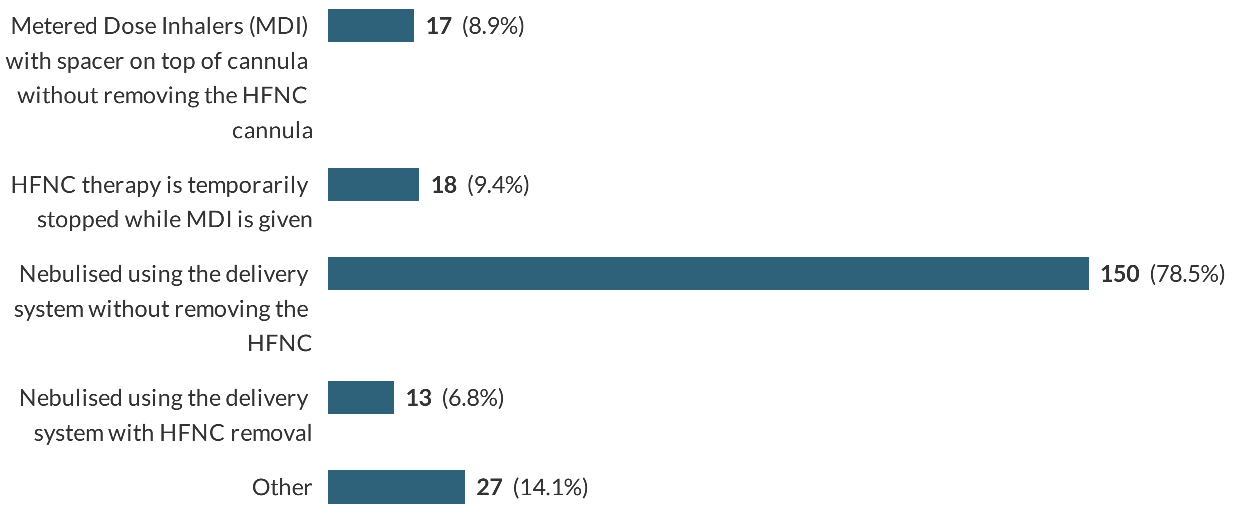

Multi answer: Percentage of respondents who selected each answer option (e.g. 100% would represent that all this question's respondents chose that option)

24.a If you selected Other, please specify:

Showing first 5 of 25 responses	
I don' t know	377609-377600-39029280
we would like to use the in line delivery system but do not have the kit.	377609-377600-39117089
in bronchiolitis dont use nebulisers	377609-377600-39813106
dont know	377609-377600-44425584
via attachment for small cannulae, otherwise over the top of HFNC without removal	377609-377600-44526819

25 While a patient is on HFNC therapy, do you usually insert an NG tube to decompress the stomach?

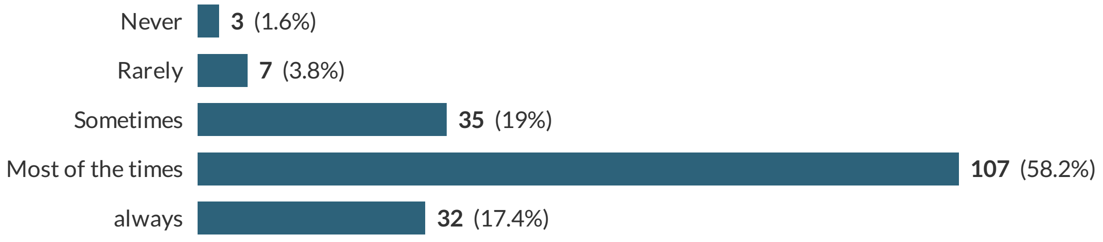

26 Regarding nutrition/feeding with HFNC therapy:

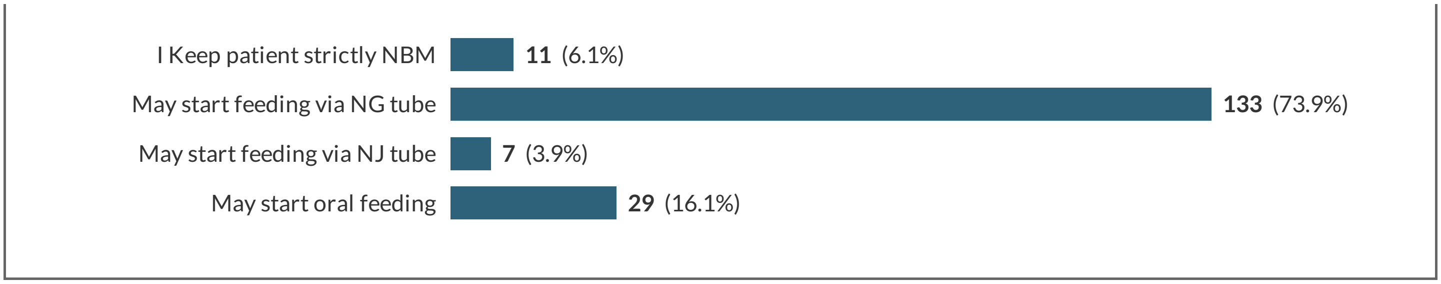

26.a If you do keep a patient NBM while on HFNC, when do you consider safe start of feed?

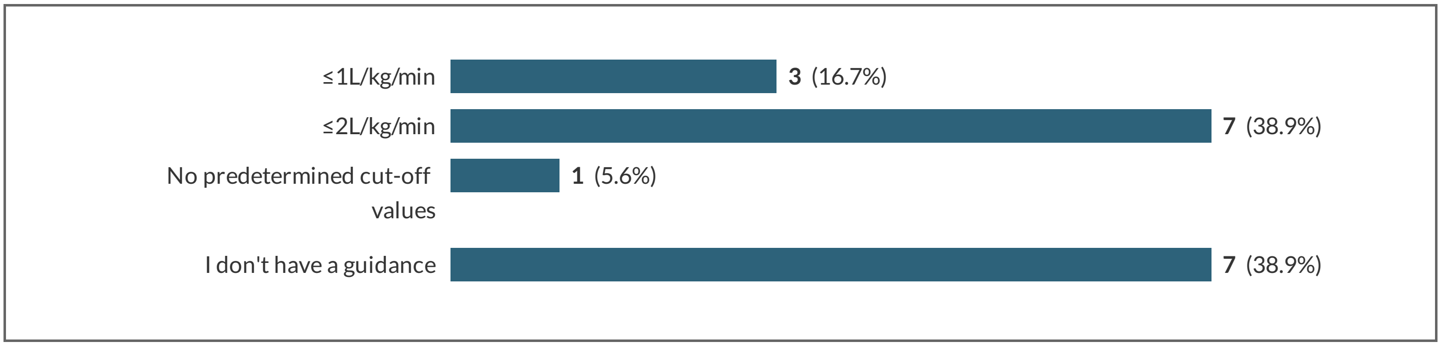

27 Do you use any type of sedation when a patient is on HFNC therapy?

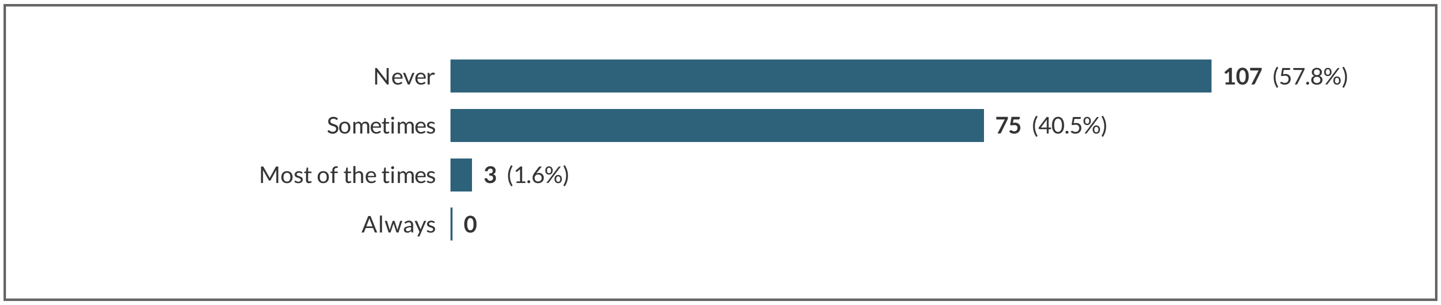

27.a If you do use sedation, what is your preferred drug?

Showing first 5 of 47 responses	
Chloral Hydrate	377609-377600-37410948
Chloral hydrate	377609-377600-39030543
Chloralhydrate	377609-377600-39039913
Chloral	377609-377600-39051526
chloral	377609-377600-39053172

28 In my opinion, in terms of "Efficacy and Clinical Effectiveness", HFNC therapy is:

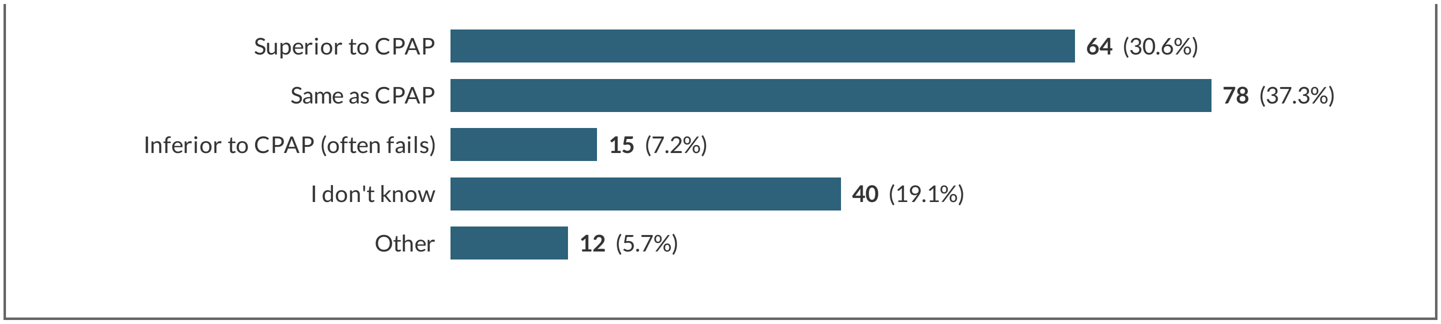

28.a If you selected Other, please specify:

Showing first 5 of 14 responses	
Depends on patient group	377609-377600-39030543
its just warm wet oxygen	377609-377600-39813106
Inferior to CPAP but more widely used	377609-377600-42628053
Too simplistic: better than CPAP in terms of ability to use without HDU capability and lower level of complications, but on the other hand not as efficacious as CPAP (though sufficiently efficacious for most patients)	377609-377600-45201715
well tolerated as compared to cpap	377609-377600-46354728

29 In my opinion, in terms of " failure and complications", HFNC therapy is:

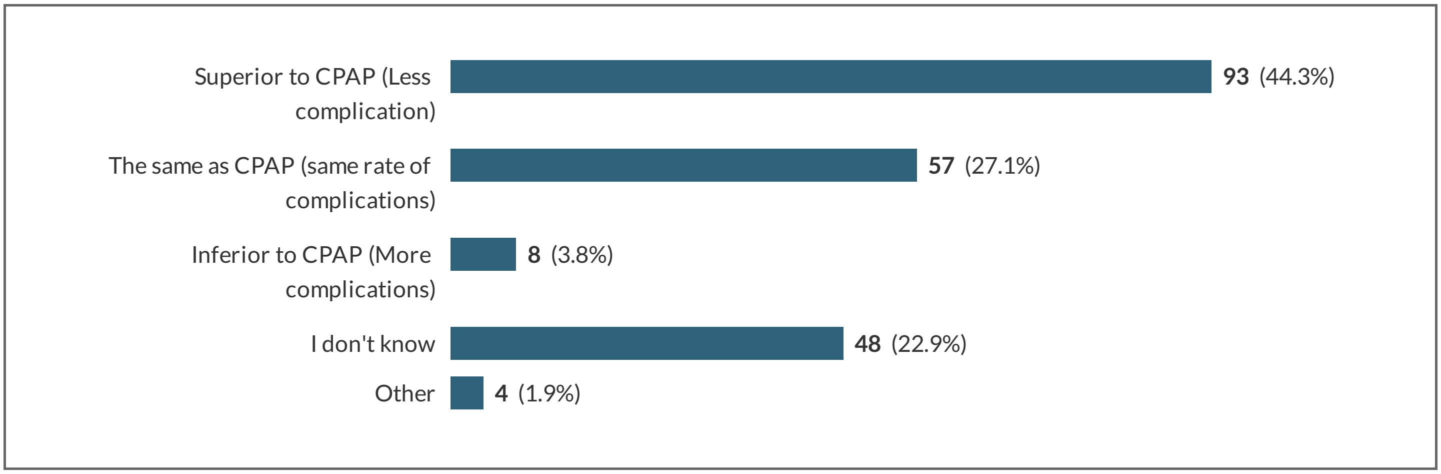

29.a If you selected Other, please specify:

Showing first 5 of 8 responses	
More likely to escalate to CPAP if fails	377609-377600-42628053
easier to set up in older children	377609-377600-47693618
May be better tolerated than CPAP, fewer harmful effects on skin around nose / face, but we have seen a case of pneumomediastinum on HFNC oxygen	377609-377600-48076511
similar but we use at a lower threshold of illness	377609-377600-48656472
I never use CPAP here	377609-377600-48990296

30 In my opinion: The THREE most important outcome measures in a Randomized Trial looking at the effect of HFNC therapy on paediatric patients: (Choose THREE: 1st, 2nd, and 3rd Most Important):

30.1 Length of Stay

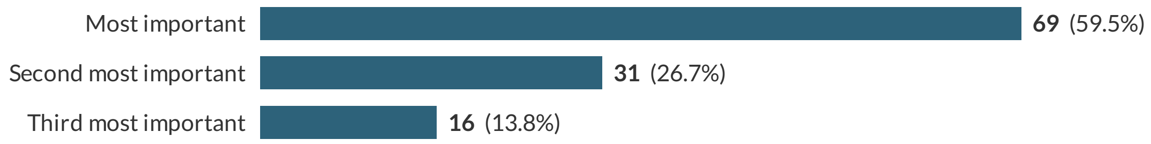

Multi answer: Percentage of respondents who selected each answer option (e.g. 100% would represent that all this question's respondents chose that option)

30.2 Length of need for HFNC support

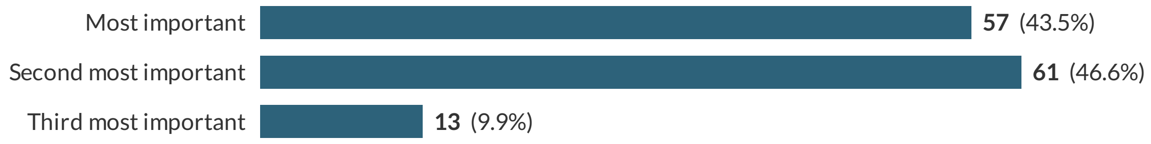

Multi answer: Percentage of respondents who selected each answer option (e.g. 100% would represent that all this question's respondents chose that option)

30.3 Failure rate (the need for other mode of NIV or invasive ventilation)

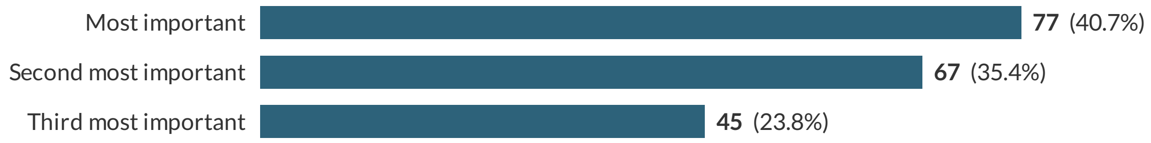

Multi answer: Percentage of respondents who selected each answer option (e.g. 100% would represent that all this question's respondents chose that option)

30.4 Length of O2 use

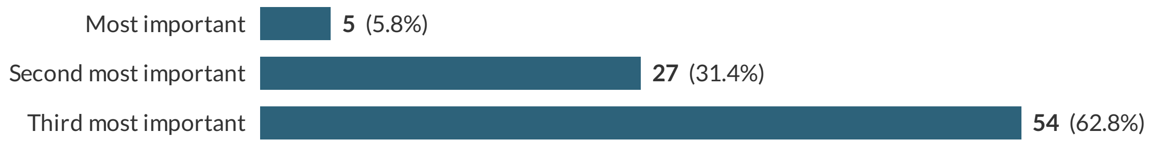

Multi answer: Percentage of respondents who selected each answer option (e.g. 100% would represent that all this question's respondents chose that option)

30.5 Cost effectiveness

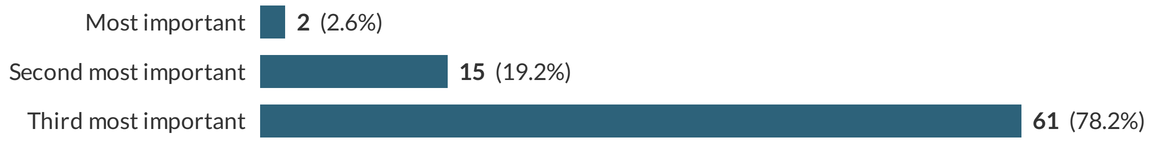

Multi answer: Percentage of respondents who selected each answer option (e.g. 100% would represent that all this question's respondents chose that option)

30.6 Patient satisfaction

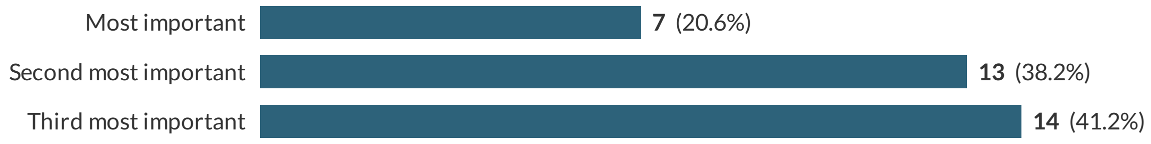

Multi answer: Percentage of respondents who selected each answer option (e.g. 100% would represent that all this question's respondents chose that option)

30.7 Parental satisfaction

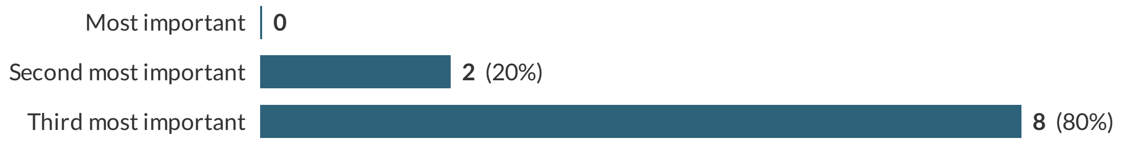

Multi answer: Percentage of respondents who selected each answer option (e.g. 100% would represent that all this question's respondents chose that option)

30.8 Other

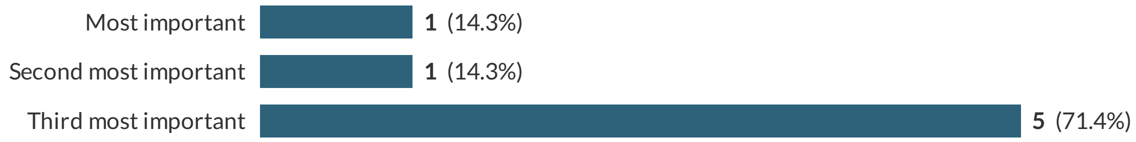

Multi answer: Percentage of respondents who selected each answer option (e.g. 100% would represent that all this question's respondents chose that option)

30.a If Other, please provide details:

**Showing all 5 responses**	
comparison to low flow o2 on length of stay and failure rate	377609-377600-39813106
avoidance of picu admission	377609-377600-45874344
length of ITU stay	377609-377600-46485120
I would combine parent/patient satisfaction as many patients opinions will be their parents opinions due to age. If patient old enough their view needs comsideration	377609-377600-46862936
Comparison with cpap for length of stay and time needed .	377609-377600-49313965

31 Do you think a national protocol on the use of HFNC therapy in the general paediatric practice would be more suitable than local protocols?

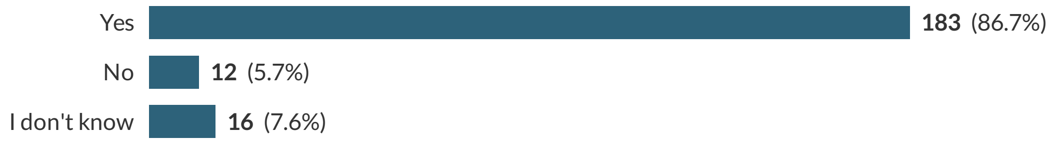

32 Thank you for taking the time completing this survey: would you like to add any comments?

Showing first 5 of 39 responses	
None	377609-377600-37410948
I've had to answer as per manufacure's protocol for some questions as our protocol uses a specified amount of oxygen not a number of litres per kg	377609-377600-39030543
In my opinion the evidence base is poor, the indications for HFO have crept upward and it is often used for respiratory distress unnecessarily, and weaned too slowly/left in situ longer than needed. However it does tend to reduce patient distress and therefore improve experience?? I put parent experience over patient experience because most patients are infants...	377609-377600-39035600
I would have answered move to CPAP to questions 18 and 21. I am not a fan of HFNC, to me this is nasal prong O2. The nose humidifies and warms inspired air so what benefit does HFNC offer? The bronchiolitis trials support the concept that HFNC is an expensive form of nasal prong O2.	377609-377600-39029280
Very important area to have more evidence in	377609-377600-39051526

Figure 1. Initiation of HHFNC and modification:

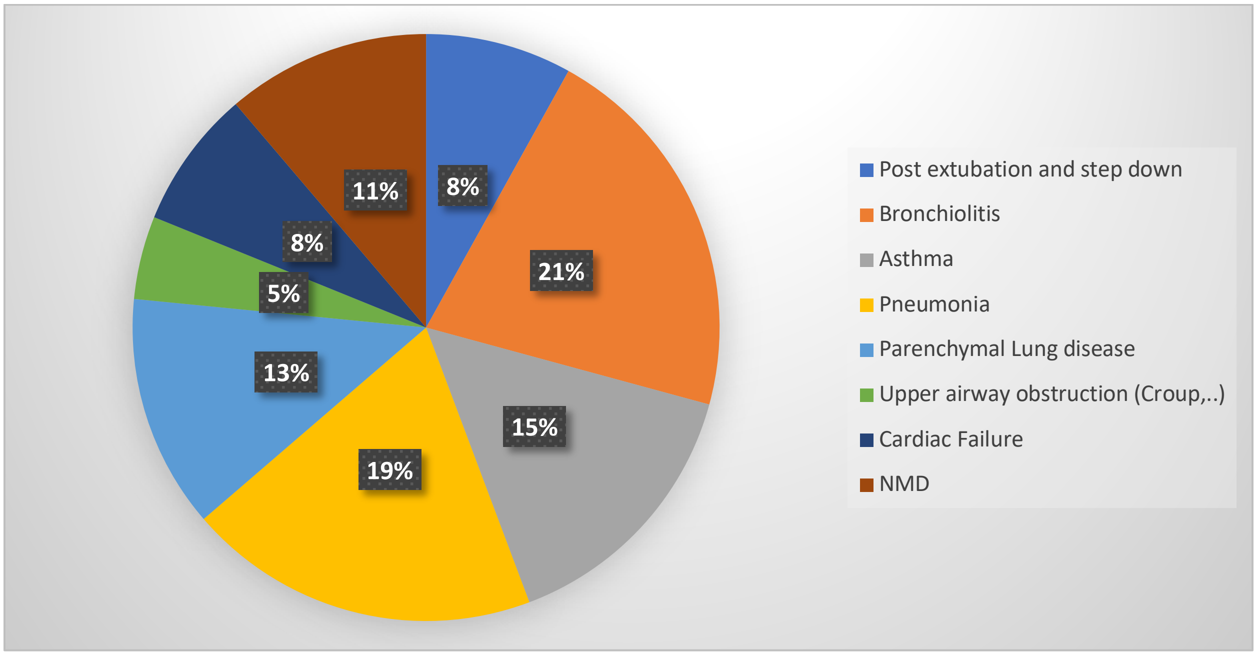

NMD: Neuromuscular diseases.

Figure 2: Indications for HHFNC on the ward:

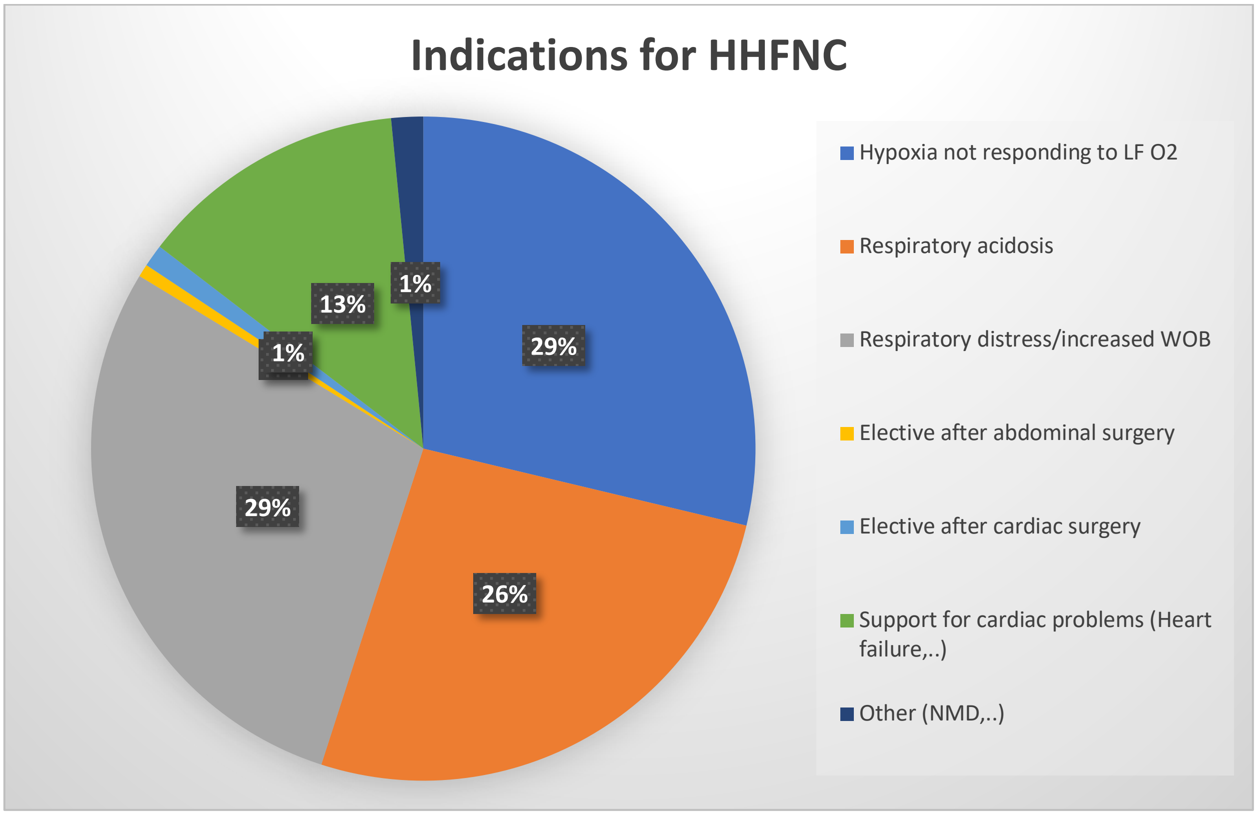

Figure 3: HHFNC in clinical practice on paediatric wards based on age and weight:

Scenario	*1- an infant (<10 kg) with moderate respiratory distress:*	*2- A child (> 10 kg) with moderate respiratory distress:*
A: What is the Start-up flow rate?	Most respondents indicated a starting flow rate for HHFNC at 2L/kg/min (76.6%)	68.9% of the respondents said they would start at 2L/kg/min
B: What is the HHFNC maximum flow rate?	35% of the respondents indicated keeping the HHFNC flow rate at 2L/kg/min, while around 40% said they could go up to 3-4 L/kg/min.	Responses were almost equal (one third each) in terms of either staying at 2L/kg/min, trying 3L/kg/min or considering the Manufacturer's recommendations if available.
C: What is the action plan if no clinical improvement while on HHFNC?	More than 70% said they will refer to the intensive care/retrieval team for further advice.	More than 70% said they will discuss the case with the intensive care/retrieval team for further advice.
Comments:	A few respondents stated an individualized protocol such as fixed flow rate regardless of the weight, or a flow range based on the weight (e.g. < 4kg: 5-8L/min, 4-10 Kg: 8-12L/min).	A few responses suggested an individualized protocol starting at 2L/Kg/min for the 1st 10 kg followed by 0.5 L/kg/min thereafter.

Figure 4: Identified Research Priorities (numbers represent responses of total 218):

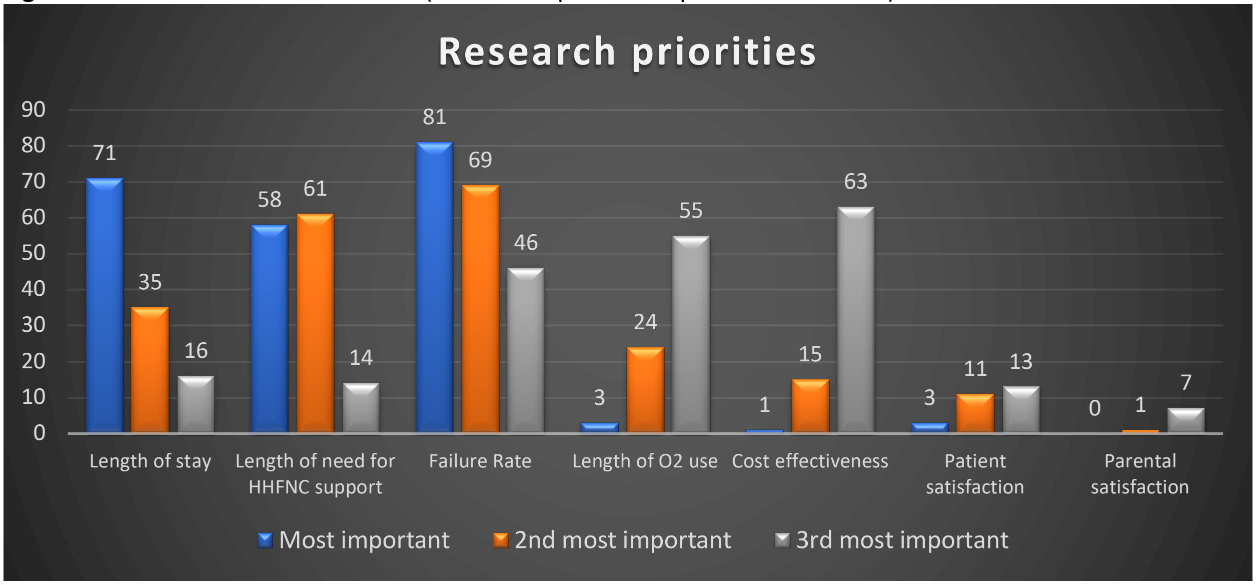

Supplement: Supplementary file 1 — Additional file 1. Survey questionnaire. [file 12887_2020_1998_MOESM1_ESM.pdf]
